# Alterations of lipid-related genes during anti-tuberculosis treatment: insights into host immune responses and potential transcriptional biomarkers

**DOI:** 10.3389/fimmu.2023.1210372

**Published:** 2023-10-31

**Authors:** Nguyen Ky Phat, Nguyen Tran Nam Tien, Nguyen Ky Anh, Nguyen Thi Hai Yen, Yoon Ah Lee, Hoang Kim Tu Trinh, Kieu-Minh Le, Sangzin Ahn, Yong-Soon Cho, Seongoh Park, Dong Hyun Kim, Nguyen Phuoc Long, Jae-Gook Shin

**Affiliations:** ^1^ Department of Pharmacology and PharmacoGenomics Research Center, Inje University College of Medicine, Busan, Republic of Korea; ^2^ Center for Personalized Precision Medicine of Tuberculosis, Inje University College of Medicine, Busan, Republic of Korea; ^3^ School of Mathematics, Statistics and Data Science, Sungshin Women’s University, Seoul, Republic of Korea; ^4^ Center for Molecular Biomedicine, University of Medicine and Pharmacy at Ho Chi Minh, Ho Chi Minh, Vietnam; ^5^ Data Science Center, Sungshin Women’s University, Seoul, Republic of Korea

**Keywords:** tuberculosis, lipid-related gene, transcriptomic biomarker, treatment monitoring, differential diagnosis

## Abstract

**Background:**

The optimal diagnosis and treatment of tuberculosis (TB) are challenging due to underdiagnosis and inadequate treatment monitoring. Lipid-related genes are crucial components of the host immune response in TB. However, their dynamic expression and potential usefulness for monitoring response to anti-TB treatment are unclear.

**Methodology:**

In the present study, we used a targeted, knowledge-based approach to investigate the expression of lipid-related genes during anti-TB treatment and their potential use as biomarkers of treatment response.

**Results and discussion:**

The expression levels of 10 genes (*ARPC5*, *ACSL4*, *PLD4*, *LIPA*, *CHMP2B*, *RAB5A*, *GABARAPL2*, *PLA2G4A*, *MBOAT2*, and *MBOAT1*) were significantly altered during standard anti-TB treatment. We evaluated the potential usefulness of this 10-lipid-gene signature for TB diagnosis and treatment monitoring in various clinical scenarios across multiple populations. We also compared this signature with other transcriptomic signatures. The 10-lipid-gene signature could distinguish patients with TB from those with latent tuberculosis infection and non-TB controls (area under the receiver operating characteristic curve > 0.7 for most cases); it could also be useful for monitoring response to anti-TB treatment. Although the performance of the new signature was not better than that of previous signatures (i.e., RISK6, Sambarey10, Long10), our results suggest the usefulness of metabolism-centric biomarkers

**Conclusions:**

Lipid-related genes play significant roles in TB pathophysiology and host immune responses. Furthermore, transcriptomic signatures related to the immune response and lipid-related gene may be useful for TB diagnosis and treatment monitoring.

## 1 Introduction

Tuberculosis (TB) is a severe infectious disease that remains a global public health emergency. According to the World Health Organization (WHO), approximately 1.4 million people lost their lives due to TB in 2022 (1). Despite attempts to reduce the duration of TB treatment, the 6-month regimen is still widely used for drug-susceptible TB (2, 3). This prolonged duration can lead to poor treatment adherence (4, 5) and consequently lead to negative outcomes such as treatment failure, relapse, antibiotic resistance, and disease spread (6, 7). Furthermore, conventional TB diagnosis and treatment monitoring rely on sputum-based tests, which have low sensitivity and modest specificity. Additionally, the collection of sputum samples for assays is difficult (8, 9). Underdiagnosis and insufficient treatment monitoring hinder the timely and accurate treatment of TB, leading to poor outcomes. Over the past two decades, significant efforts have been made to develop novel non–sputum-based biomarkers that can be used to rapidly and accurately identify active TB infection and monitor the treatment response (10). Among these biomarkers, blood transcriptomic biosignatures, which reflect host immune responses during anti-TB treatment, are promising candidates (11).

Although multiple transcriptomic signatures for the diagnosis of TB have been proposed (12, 13), the dynamic responses of these biomarkers to TB treatment have not been the main focus in prior works. Only a few transcriptomic signatures have been evaluated for use in the monitoring of anti-TB treatment (9, 10, 14). These signatures have also been found to be useful for TB diagnosis, treatment monitoring, and risk prediction (9). A multi-national study validated the use of the six-gene RISK6 signature (*TRMT2A*, *SDR39U1*, *TUBGCP6*, *SERPING1*, *GBP2*, and *FCGR1B*) for TB diagnosis and treatment monitoring. This signature had a high performance for the differentiation of untreated patients with those who completed the intensive phase of treatment, at the end of treatment, and those who had completed treatment two months previously. Additionally, the RISK6 signature fulfills the WHO target product profile criteria for screening/triage tests for the diagnosis of TB (15). We previously developed the Long10 signature comprising 10 genes (*CD274*, *KIF1B*, *IL15*, *TLR1*, *TLR5*, *FCGR1A*, *GBP1*, *NOD2*, *GBP2*, *EGF*) that were consistently downregulated during TB treatment. The signature displayed comparable performance to other signatures for TB diagnosis, treatment monitoring, and risk assessment (16). The satisfactory performance of the RISK6 and Long10 signatures suggests that a combination of transcriptomic biosignatures can be useful for multiple aspects of TB management. Although the Sambarey10 signature (*FCGR1A*, *HK3*, *RAB13*, *RBBP8*, *IFI44L*, *TIMM10*, *BCL6*, *SMARCD3*, *CYP4F3*, *SLPI*) showed promising performance in TB diagnosis, it has not been evaluated for the monitoring of treatment responses (12, 13, 17). Furthermore, in an individual participant data meta-analysis, only the Sambarey10 and Sweeney3 signatures fulfilled the WHO target product profile criteria for TB triage tests, which requires 90% sensitivity and 70% specificity at the minimum (13, 18).

Despite significant advancements in recent decades, further efforts are needed to develop transcriptional biomarkers for use in TB management. In a prospective cohort study, none of the evaluated transcriptome-based biosignatures fulfilled the WHO target product profile criteria for blood-based confirmatory tests (9). The significant variations in host responses to TB among individuals, cohorts, and comorbidities make the development of a universal biosignature challenging (11). Additionally, the multi-step experimental process, statistical analysis pipelines for data with thousands of genes, and the nature of array- or next-generation sequencing-based technologies can lead to high false-positive rates (19–23). Thus, the reproducibility and robustness of biosignatures need to be improved.

Lipid signaling and immune responses are complex, interlinked processes. Lipoproteins, free fatty acids (FFAs), lipokines, interleukins, and other biological components modulate the complex interactions between these systems (24). In TB, proinflammatory lipid signaling cascades are associated with tricarboxylic acid cycle remodeling, increased interleukin-1β expression, and decreased granulocyte-macrophage colony-stimulating factor expression (25). Our previous study showed significant perturbations related to metabolism and immune response of the host signaling based on the alteration in plasma lipid profiles between TB patients and non-TB controls. Subsequently, dysregulated metabolic and signaling pathways were identified using gene enrichment analysis. Among the genes involved in these pathways, 162 non-overlapped lipid-related genes potentially associated with the pathophysiology of TB were extracted and validated in three datasets (26). Our other study of the plasma lipidome of patients with TB during the 6-month treatment regimen showed changes in pathways related to lipid metabolism and the host immune response (27). These findings suggest an association between systemic lipid alterations and TB disease status. Thus, changes in lipid-related genes may serve as indicators of the response to TB treatment.

**Figure 1:**
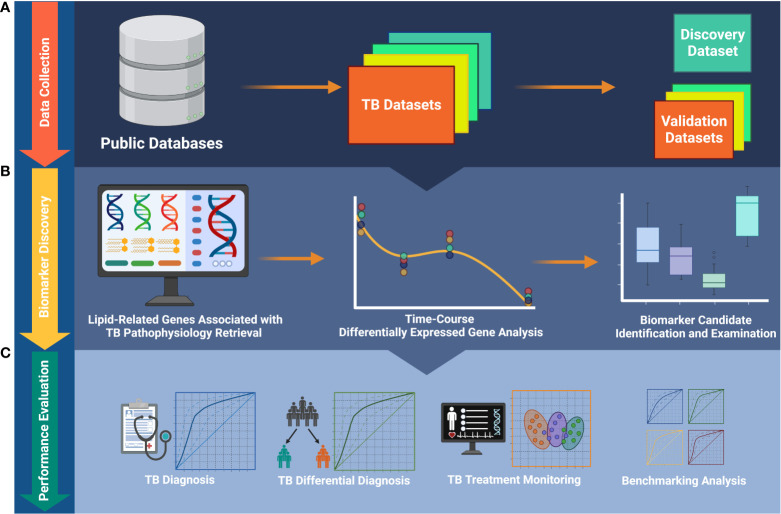
The workflow of the study. **(A)** Publicly available data collection. **(B)** Identification of biomarkers and foundation of biosignature. **(C)** Validation of the ability of the biosignature for TB diagnosis and treatment monitoring. TB, tuberculosis.

In the present study, we used a targeted, knowledge-based approach to select the most significant TB biomarker candidates from the 162 lipid-related genes previously found. The study workflow is described in **Figure 1**. We evaluated the potential usefulness of these genes for pulmonary TB diagnosis and treatment monitoring in multiple cohorts. Additionally, we conducted a benchmark analysis to compare the performance of the candidate biomarkers with that of publicly available signatures. We found that the performance of the lipid-related genes was not better than that of certain other biosignatures. Our results demonstrate that lipid metabolism is involved in the host immune response during TB treatment. Importantly, we provide more evidence that lipid metabolism and signaling researches can contribute to improve the management of TB.

## 2 Materials and methods

### 2.1 Published transcriptomics data acquisition

Transcriptomic datasets of pulmonary TB were collected from the Gene Expression Omnibus (GEO) and ArrayExpress databases. The search term was built as previously described and restricted to *Homo sapiens* species (16). For longitudinal datasets, drug susceptibility (DS)-TB cases with no known severe comorbidities were selected. Additionally, patients with known failure treatment outcomes were excluded. Three representative datasets [GSE31348 (28), GSE89403 (29), and GSE181143 (30)] were included for subsequent analyses to demonstrate the dynamic response of lipid-related genes during the TB treatment time course. These three longitudinal TB datasets were obtained from patients who underwent the standard six-month anti-TB treatment. GSE31348 was used as the identification cohort, while GSE89403 and GSE181143 were utilized for validation. Additional datasets were collected to demonstrate the potential of lipid-related genes in TB diagnosis. They covered different medical conditions with or without human immunodeficiency virus (HIV), including TB, latent TB infection (LTBI), non-TB, and other diseases (OD). Of note, the OD groups from the GSE37250 dataset consist of patients with multiple diseases that are common in the African population (e.g., pneumonia (PNA)/lower respiratory tract infection/*Pneumocystis jirovecii* pneumonia; malignancy and other neoplasia other than Kaposi’s sarcoma; pelvic inflammatory disease/urinary tract infection; bacterial, viral meningitis, or meningitis of uncertain origin; and hepatobiliary disease). Detailed information is available in the original study of the dataset (31). The collected datasets were also comprised of healthy control, active sarcoidosis (SARC), non-active SARC, lung cancer, and pneumonia individuals. Eight chosen datasets were E-MTAB-8290 (non-TB-non-HIV, non-TB-HIV, TB, TB-HIV) (32), GSE37250 (OD, OD-HIV, LTBI, LTBI-HIV, TB, and TB-HIV) (31), GSE107991 (healthy control, TB, LTBI) (33), GSE107994 (healthy control, TB, LTBI) (33), GSE101705 (TB, LTBI) (34), and a combined dataset from GSE42825, GSE42826, and GSE42830 (healthy control, active SARC, non-active SARC, lung cancer, PNA) (35). The information of all datasets included in this study is summarized in **Supplementary Table 1**.

### 2.2 Targeted lipid-related genes list and other available signatures

A list of 162 lipid-related genes associated with the biological pathways underlying TB pathophysiology was retrieved from our previous study (**Supplementary Table 2**) (26). This list was extracted from significantly enriched pathways of our reported lipid biomarkers for TB and non-TB control differentiation. Of note, all of these 162 genes are host genes and not derived from *Mycobacterium tuberculosis* (*Mtb*).

For comparison purposes, three other signatures were created by directly extracting the component genes of publicly available signatures, i.e., RISK6 (36), Sambarey10 (17), and Long10 (16).

### 2.3 Data processing and normalization

Microarray data were normalized using *affy* (version 1.74.0, Affymetrix) (37) and *lumi* (version 2.48.0, Illumina) (38) packages, respectively. The batch effects of microarray datasets with multi-site cohorts was corrected by the Combat method (39) using *sva* package (version 3.44.0) (40) after being examined with the *BatchQC* package (version 1.24.0) (41). Regarding RNA-seq data, the batch effect of datasets was inspected by *BatchQC* and corrected using Combat-seq (42). RNA-seq data were normalized using the median of ratio method combined with regularized logarithmic transformation. The pipeline was conducted by the *DESeq2* package (version 1.36.0) (43).

### 2.4 Single-sample scoring of gene signature

Gene set variation analysis (GSVA) was carried out using *GSVA* package (version 1.44.5) (44) to evaluate the treatment monitoring and diagnosis characteristics of gene signatures. GSVA transforms the transcriptome profile of an individual sample into a signature enrichment profile. The GSVA score of a signature characterizes the coordination in the regulation (either up or down) of its component genes and indicates its activity level.

### 2.5 Statistical analysis

The molecular profiles of the lipid-related genes of patients during TB treatment were examined using principal component analysis (PCA) and t-distributed stochastic neighbor embedding (t-SNE). The profiles were visualized using three-dimensional PCA and t-SNE score plots drawn by *plotly* package (version 4.10.1). *ComplexHeatmap* package (version 2.15.1) (45) was used to create heatmap visualization of GSVA score for obtained biosignature. A polynomial regression targeted to 162 lipid-relate genes was implemented by *maSigPro* package (version 1.68.0) (46) to identify differentially expressed genes (DEGs) during the TB treatment time course. In short, the algorithm built a profile model for time-course gene expression:


$$y_{i}={\text{β}}_{0}+{\text{β}}_{1}{\text{T}}_{i}+\;{\text{β}}_{1}{\text{T}}_{i}^{2}+\ldots +\;{\text{β}}_{d}{\text{T}}_{i}^{d}+{\text{η}}_{1}{\text{Z}}_{i1}+\ldots {\text{η}}_{\text{p}}{\text{Z}}_{\text{ip}}+\epsilon_{i,}$$

where y*_i_* is the expression level for a gene and $\epsilon_{i}$ is the error term. The model consists of two parts: (1) polynomial of degree *d* in the time variable and (2) the linear regression explained by *p* explanatory variables. This is assumed to be the full model, but due to model complexity, the package considers a reduced model, which uses fewer variables than the full model but still has enough predictive power. The selection procedure is either forward or backward step-wise selection where each variable is sequentially tested if the addition or elimination of the variable improves the model. In our case, we did not include any explanatory variable and set the polynomial degree as 2. For testing the overall significance of the regression model, F-test was performed for each gene. Lipid-related genes with a false discovery rate (FDR) < 0.05 were selected as DEGs. The *R^2^* value was set to ≥ 0.3 for selecting the genes as biomarker candidates.

The Kruskal-Wallis test and the *post hoc* two-sided unpaired Wilcoxon rank sum test were performed for testing unpaired data. With paired data, the Friedman test followed by the *post hoc* paired Wilcoxon signed rank test was applied. For a single statistical testing, a raw P-value < 0.05 was considered statistically significant. For multiple comparisons, the FDR of 0.05 was established as the significant threshold. Statistical tests were conducted using *rstatix* package (version 0.7.1).

### 2.6 Gene signature performance evaluation

The potential of the signature to characterize different TB treatment states was evaluated by applying k-means clustering. In detail, the expression profile of each gene was considered a variable, and the sample at a specific time point was treated as an observation. The number of clusters was predetermined. As a result, the algorithm identified clusters of samples that exhibited similar expression profiles of the signature, regardless of their actual sampling time point. The data was Pareto scaled prior to the analysis. MetaboAnalyst 5.0 (47) was employed to conduct the classification model.

For TB diagnosis, the classification model was built using a logistic regression. Model validation was performed with a 10-fold nested cross-validation procedure where the outer loop is for splitting training and test data and the inner for searching the best tuning parameters. The *caret* package (version 6.0-93) (48) was used for model building and validation. Model performance was assessed by the area under the curve (AUC) value of the receiver operating characteristic (ROC) curve. All statistical analyses and presentations were implemented in R version 4.2.1. Besides the base R graphics, the *ggplot2* (version 3.4.0) and its extension *ggpubr* (version 0.5.0) were used for visualization unless stated otherwise.

## 3 Results

### 3.1 The association of 162 lipid-related genes with different TB treatment states and various disease conditions

**Figure 2:**
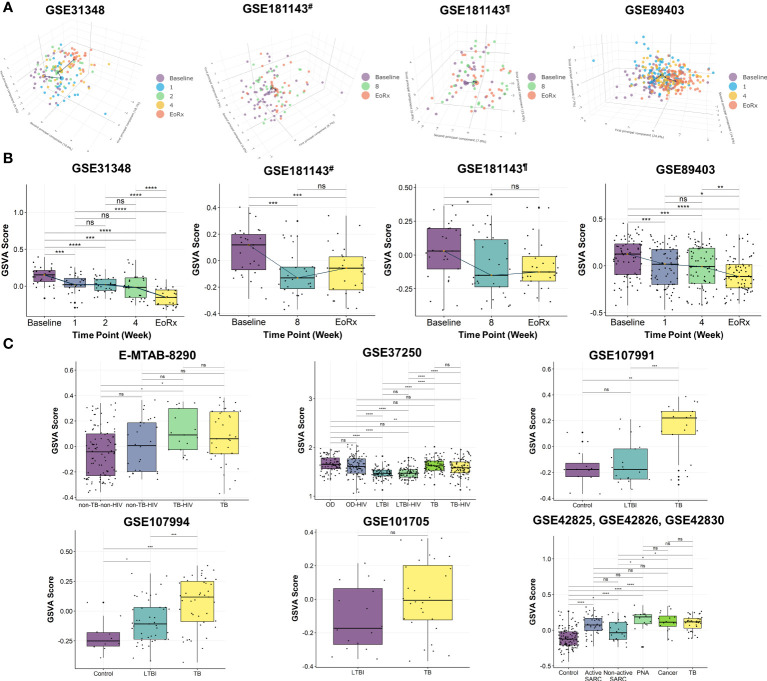
The potential of 162 lipid-related genes in TB treatment monitoring and diagnosis. **(A)** 3D principal component analysis scores plots represent the transcriptome profiles for 162 lipid-related genes during the TB treatment. **(B)** The GSVA score of 162 lipid-related genes across the TB treatment time course. The orange point represents the median GSVA score of the subject group and the box plot represent the correspondent interquartile range. **(C)** The GSVA score of 162 lipid-related genes in TB and its counterparts. GSVA, gene set variation analysis; 1, after one week; 2, after two weeks; 4, after four weeks; 8, after eight weeks; EoRx, treatment completion; TB, tuberculosis; LTBI, latent TB infection; OD, other diseases; HIV, human immunodeficiency virus; SARC, sarcoidosis; Cancer, lung cancer; PNA, pneumonia; #, subset from India of GSE181143 dataset; ¶, subset from Brazil of GSE181143 dataset; ns, not significant; *,<0.05; **,<0.01; ***,<0.001, ****,<0.0001; *two-sided paired Wilcoxon signed rank test* **(B)** and *two-sided Wilcoxon rank sum test* **(C)**.

PCA was performed to investigate the dynamic responses of the expression profiles of 162 lipid-related genes during TB treatment. The PCA scores plots showed clear separations between the transcriptomes at baseline and at treatment completion, but not after one, two, four, or eight weeks of treatment, in all datasets except the Brazilian population subset of GSE181143 (no clear separation was observed for this subset) (**Figure 2A**). Interestingly, t-SNE displayed three clusters of lipid-related gene transcriptomes (*i.e.*, baseline, mid-time points, and treatment completion) as observed with PCA, except GSE181143 cohort (**Supplementary Figure 1**). The lipid-related gene expression profiles generally formed three clusters corresponding to baseline, treatment completion, and other time points.

To further explore the associations between alterations in the gene expression of lipid-related genes and TB treatment states, we performed GSVA. The GSVA score declined significantly from baseline to treatment completion, reflecting that the lipid-related genes were less activated at the end of the treatment course (**Figure 2B**). Moreover, the Friedman test demonstrated significant differences in GSVA scores across various time points in GSE31348, GSE89403, and GSE181143 (**Supplementary Table 3**). Pairwise comparison showed that the GSVA score for the 162 lipid-related genes differed significantly between baseline, mid-treatment, and treatment completion, with no significant difference among mid-treatment time points. In GSE181143, no significant difference was observed between the mid-treatment (after eight weeks) time point and treatment completion. These findings were in line with the PCA results.

We also performed GSVA to investigate the association between the lipid-related genes and different disease conditions. The GSVA score was significantly higher for patients with TB than for those with other conditions (i.e., non-TB-non-HIV, LTBI, LTBI-HIV, healthy control, non-active SARC) (**Figure 2C**) and differed significantly across patient groups. However, no significant differences were found between the TB group with OD, active SARC, lung cancer, and PNA groups (**Figure 2C**, **Supplementary Table 4**). Additionally, the GSVA score of the LTBI group differed significantly from those of other groups (GSE37250 and GSE107994). Notably, the GSVA score did not differ significantly between patients with and without HIV infection in the non-TB (E-MTAB-8290), OD, LTBI, or TB groups (GSE37250). Taken collectively, these results indicated the association between 162 lipid-related genes and TB treatment states. This suggested further investigation into the ability of 162 lipid-related genes for TB treatment monitoring and diagnosis.

### 3.2 The foundation of 10-lipid-gene transcriptional signature

To develop a clinically applicable biosignature, 162 genes were screened to identify the most promising candidate biomarkers. A time-course regression analysis targeted to 162 lipid-related genes was conducted on 135 samples with 5 different time points (GSE31348) to identify DEGs throughout TB treatment. The analysis identified 80 lipid-related genes that are differentially expressed during TB treatment, with most changes in the expression levels of these genes were subtle. Ten DEGs with *R^2^* > 0.3 were identified as the most potential biomarker candidates (**Table 1**). The ten lipid-related genes together formed the so-called “10-lipid-gene” signature.

**Table 1:** List of the most potential lipid-related gene candidates.

EntrezID	Gene Symbol	Gene Name	FDR*	R-squared*	Main Biological Function of Encoded Protein	Reference
10092	*ARPC5*	actin-related protein 2/3 complex subunit 5	5.23E-11	0.335	Regulate fatty acid synthesis. Involve in cup formation during phagocytosis Regulate the homeostasis of T cells	(49–51)
2182	*ACSL4*	acyl-coenzyme A synthetase long-chain family member 4	1.74E-11	0.349	Promote fatty acid oxidation and lipid biosynthesis Regulate ferroptosis	(52, 53)
122618	*PLD4*	phospholipase D family member 4	5.52E-13	0.396	Involve in macrophage activation and phagocytosis	(54, 55)
3988	*LIPA*	lipase A, lysosomal acid type	1.74E-11	0.348	Generate free fatty acids and free cholesterol Involves in the maturation and function of immune cells	(56)
25978	*CHMP2B*	charged multivesicular body protein 2B	5.45E-10	0.307	Participate in membrane remodeling and repair Involves in fatty acid trafficking to maintain lipid and energy homeostasis	(57, 58)
5868	*RAB5A*	RAB5A, member RAS oncogene family	1.74E-11	0.349	Encode a small GTPase that regulates endocytosis	(59, 60)
11345	*GABARAPL2*	gamma-aminobutyric acid receptor-associated protein-like 2	3.32E-10	0.315	Regulate lipid droplet biogenesis Facilitate autophagosome formation	(61, 62)
5321	*PLA2G4A*	phospholipase A2 group IVA	6.05E-10	0.305	Regulate lipid droplet biogenesis Participate in initial step of the arachidonic acid pathway	(63, 64)
129642	*MBOAT2*	membrane-bound O-acyltransferase domain containing 2	1.23E-11	0.360	Regulate the free arachidonic acid level through arachidonate recycling process	(65)
154141	*MBOAT1*	membrane-bound O-acyltransferase domain containing 1	4.68E-10	0.310	Regulate the free arachidonic acid level through arachidonate recycling process	(65)

FDR, False Discovery Rate.

*FDR and R-squared values were obtained from time series analysis using GSE31348.

**Figure 3:**
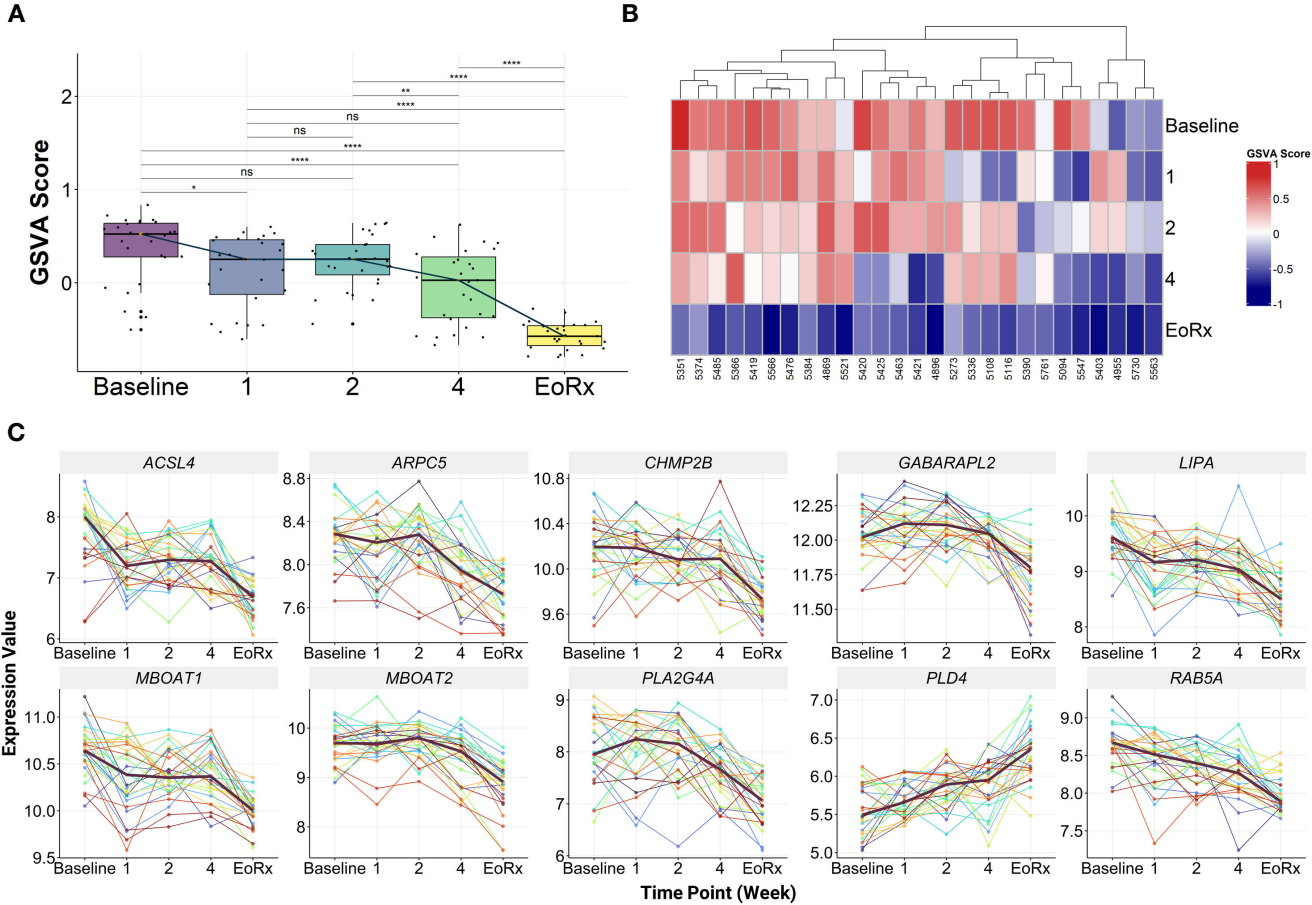
The characteristics of 10-lipid-gene biosignature. **(A)** The GSVA score of 10-lipid-gene signature during TB treatment (N = 27). The orange point represents the median GSVA score of the subject group and the box plot represent the correspondent interquartile range. **(B)** Heatmap represents the signature enrichment profiles of individual patients for 10-lipid-gene signature during TB treatment (N = 27). **(C)** The expression level of individual gene component of the signature across the TB treatment time course (N = 27). The dark mauve line indicates the median gene expression level. GSVA, gene set variation analysis; 1, after one week; 2, after two weeks; 4, after four weeks; EoRx, treatment completion; ns, not significant; *,<0.05; **,<0.01; ****,<0.0001; *two-sided paired Wilcoxon signed rank test*.

GSVA was performed on the discovery dataset (i.e., GSE31348) to demonstrate the dynamic response of the 10-lipid-gene signature. Overall, the GSVA scores for the signature showed similar changes during treatment as did those for the 162 lipid-related genes (**Figures 2B, 3A**, **Supplementary Table 5**). GSVA scores for all 27 patients of the discovery dataset were visualized using heatmaps to examine the interindividual response variability in the 10-lipid-gene signature. Although some patients showed unusual patterns of change during the initial four weeks of treatment, most cases showed a significant reduction in the GSVA score at treatment completion (**Figure 3B**). The changes in individual gene expression of all 27 patients in the GSE31348 dataset were analyzed to determine the gene-specific variability. Two main trends were observed during TB treatment: chronological down-regulation and up-regulation (**Figure 3C**). In particular, the expression of nine genes (*ARPC5*, *ACSL4*, *LIPA*, *CHMP2B*, *RAB5A*, *GABARAPL2*, *PLA2G4A*, *MBOAT2*, and *MBOAT1*) was altered only slightly during the initial four weeks of treatment, but was down-regulated significantly at treatment completion. In contrast, the expression of *PLD4* increased during treatment.

### 3.3 The ability of 10-lipid-gene transcriptional biosignature to reflect different TB treatment states

**Figure 4:**
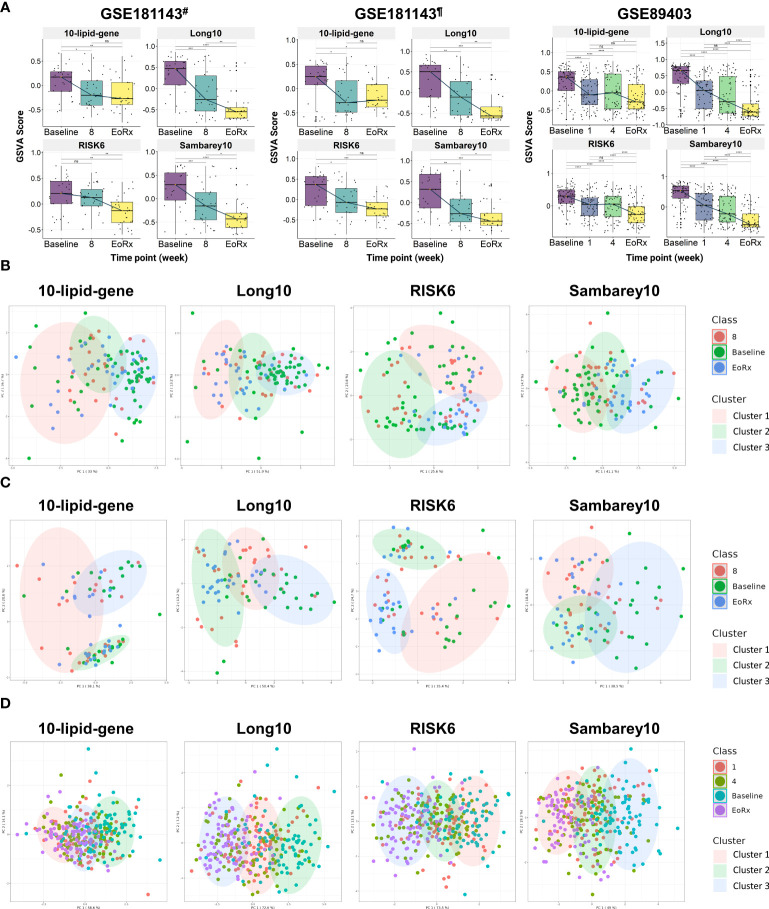
The potential 10-lipid-gene biosignature in TB treatment monitoring. **(A)** The GSVA score of 10-lipid-gene, Long10, RISK6, and Samberey10 biosignatures during the TB treatment time course. The orange point represents the median GSVA score of the subject group and the box plot represent the correspondent interquartile range **(B)** Classification of different TB treatment states based on the 10-lipid-gene signature for subset from India of the GSE181143 dataset. **(C)** Classification of different TB treatment states based on the 10-lipid-gene signature for subset from Brazil of the GSE181143 dataset. **(D)** Classification of different TB treatment states based on the 10-lipid-gene signature for the GSE89403 dataset. GSVA, gene set variation analysis; 1, after one week; 2, after two weeks; 4, after four weeks; 8, after eight weeks; EoRx, treatment completion; #, the subset of Indian samples from GSE181143 dataset; ¶, the subset of Brazilian samples from GSE181143 dataset. ns, not significant; *,<0.05; **,<0.01; ***,<0.001; ****,<0.0001; *two-sided paired Wilcoxon signed rank test*.

External validation was performed to evaluate the dynamic response of the 10-lipid-gene biosignature during TB treatment (**Figure 4A**). To enhance the reliability of the assessment, we also compared it with other priory-established signatures (i.e., Long10, RISK6, and Sambarey10). In the two subsets of GSE181143, the 10-lipid-gene signature was down-regulated from baseline to after eight weeks and remained stable until treatment completion (**Supplementary Table 6**). However, the GSVA scores for the other three signatures decreased consistently during the treatment course; this reduction was subtle for the RISK6 signature and significant at all-time points for the Long10 and Sambarey10 signatures (**Figure 4A**, **Supplementary Table 6**). In the Catalysis treatment response cohort (CTRC) (i.e., GSE89403), the GSVA scores for the 10-lipid-gene and RISK6 signatures decreased from baseline to after one week, remained stable after four weeks, and thereafter continued to decrease until treatment completion. **Figure 4A** shows the significant reduction of the GSVA scores for the Long10 and Sambarey10 signatures during treatment in the CTRC cohort. Notably, only the score for the Sambarey10 signature differed significantly between after one week and after four weeks of treatment.

We evaluated the ability of the 10-lipid-gene signature to differentiate among TB treatment states using k-means clustering. Three clusters were pre-determined to correspond to the distinct states of TB treatment (baseline, mid-time points, and treatment completion) that were previously observed. Overall, the three clusters showed a high degree of overlap in all datasets. In the subset from India of GSE181143, the 10-lipid-gene signature exhibited weak performance, with a cluster corresponding to the baseline but none corresponding to the other time points; the performance of 10-lipid-gene signature was not superior to other signatures (**Figure 4B**). In the cohort from Brazil of GSE181143, the grouping based on 10-lipid-gene signature was not in concordance with TB treatment states (**Figure 4C**). The RISK6 signature could cluster the samples at treatment completion and the Sambarey10 signature could cluster samples at baseline, although the tendency is unclear. Remarkably, the Long10 signature exhibited good concordance with the original labels in clustering the samples at baseline and at the end of treatment. In the CTRC cohort, the clusters based on the 10-lipid-gene signature and the treatment states were not concordant (**Figure 4D**). The other three signatures surpassed the 10-lipid-gene signature in clustering the samples into different TB treatment states, illustrated by better concordance between the original labels and pre-determined clusters. These findings are in line with the observation of subtle changes in the 10-lipid-gene signature during TB treatment. Collectively, the 10-lipid-gene biosignature exhibited weak clustering ability and only partially reflected the TB treatment states.

### 3.4 The ability of 10-lipid-gene transcriptional biosignature for TB diagnosis and TB differential diagnosis

**Figure 5:**
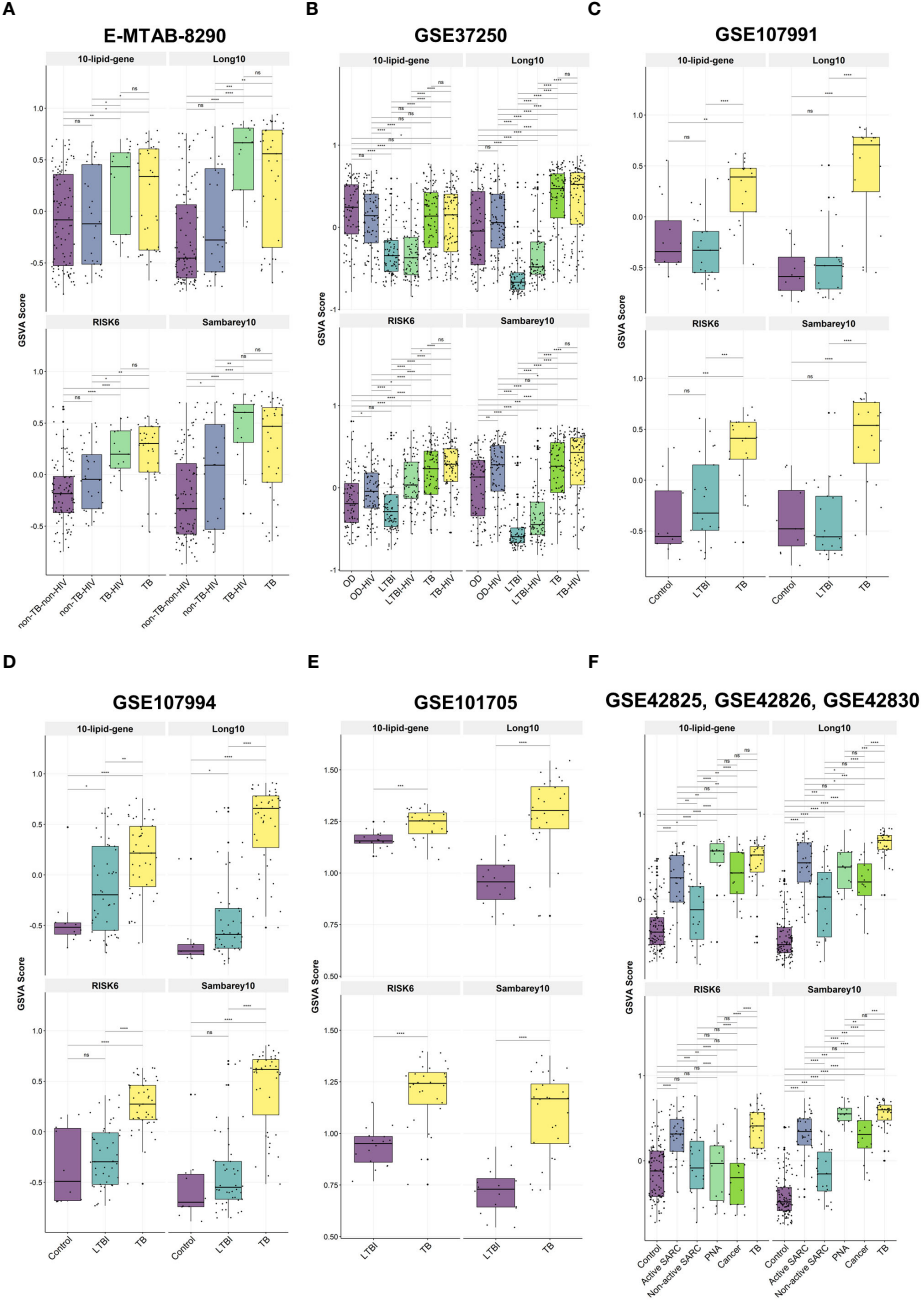
The GSVA score of 10-lipid-gene, Long10, RISK6, and Samberey10 biosignatures in TB and its counterparts. **(A)** E-MTAB-8290. The box plot represents the median and the interquartile range of the GSVA score for each subject group **(B)** GSE37250. **(C)** GSE107991. **(D)** GSE107994. **(E)** GSE101705. **(F)** GSE4285, GSE42826, GSE42830. GSVA, gene set variation analysis; TB, tuberculosis; LTBI, latent TB infection; OD, other diseases; HIV, human immunodeficiency virus; SARC, sarcoidosis; Cancer, lung cancer; PNA, pneumonia; ns: not significant; *,<0.05; **,<0.01; ***,<0.001; ****,<0.0001; *two-sided Wilcoxon rank sum test*.

We investigated the relationships between the 10-lipid-gene signature and multiple subject groups in various clinical situations. Notably, GSVA scores for this signature were higher for patients with TB than for most other groups, irrespective of the HIV status, except in the GSE37250 and the combined GSE42825/GSE42826/GSE42830 dataset (**Figures 5A–F**, **Supplementary Table 7**). The GSVA score differed significantly across subject groups. In particular, the GSVA score for the 10-lipid-gene signature differed significantly between the TB groups and the other groups (excluding the OD groups in GSE37250 as well as cancer and PNA in GSE42825, GSE42826, and GSE42830) (**Figures 5A–F**). These findings are in line with those observed for the 162 lipid-related genes (**Figures 2B, C**). Interestingly, the simplified signature showed a better ability than the 162-gene set to differentiate between TB and non-TB-HIV, TB and active SARC, and TB-HIV and non-TB-HIV groups based on the GSVA score (**Figures 2B, C**, **5A, F**). The GSVA score patterns for three other signatures were similar to that for the 10-lipid-gene signature in most datasets, except in the GSE37250 and the combined GSE42825/GSE42826/GSE42830 dataset. However, based on the GSVA scores, the other signatures showed comparable or better distinction among subject groups than the 10-lipid-gene signature. None of the tested signatures had a GSVA score that differed significantly between the TB and TB-HIV groups.

**Figure 6:**
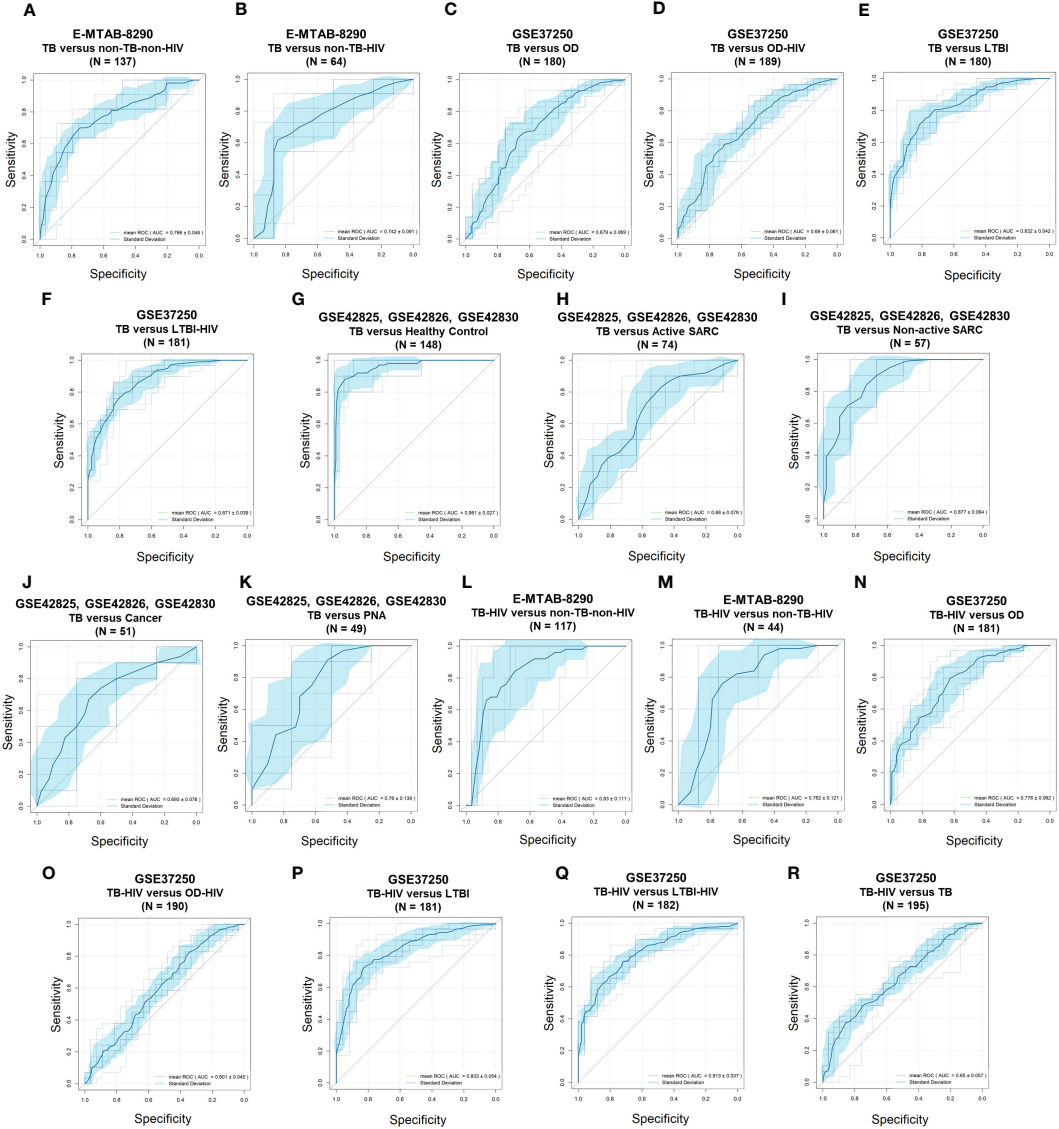
Receiver operating characteristic curves of the 10-lipid-gene signatures in TB diagnosis. **(A)** E-MTAB-8290, TB *versus* non-TB-non-HIV. **(B)** E-MTAB-8290, TB *versus* non-TB-HIV. **(C)** GSE37250, TB *versus* OD. **(D)** GSE37250, TB versus OD-HIV. **(E)** GSE37250, TB *versus* LTBI. **(F)** GSE37250, TB *versus* LTBI-HIV. **(G)** GSE42825; GSE42826; GSE42830, TB *versus* healthy control. **(H)** GSE42825; GSE42826; GSE42830, TB *versus* active SARC. **(I)** GSE42825; GSE42826; GSE42830; TB *versus* non-active SARC. **(J)** GSE42825; GSE42826; GSE42830, TB versus Cancer. **(K)** GSE42825; GSE42826; GSE42830, TB *versus* PNA, **(L)** E-MTAB-8290, TB-HIV *versus* non-TB-non-HIV. **(M)** E-MTAB-8290, TB-HIV *versus* non-TB-HIV. **(N)** GSE37250, TB-HIV versus OD. **(O)** GSE37250, TB-HIV versus OD-HIV. **(P)**, GSE37250, TB-HIV *versus* LTBI. **(Q)** GSE37250, TB-HIV versus LTBI-HIV. **(R)** GSE37250, TB-HIV versus TB. TB, tuberculosis; LTBI, latent TB infection; OD, other diseases; HIV, human immunodeficiency virus; SARC, sarcoidosis; Cancer, lung cancer; PNA, pneumonia.

The differential diagnosis performance of the 10-lipid-signature was evaluated in multiple clinical cohorts (**Figure 6**, **Table 2**). A logistic regression classifier based on the 10-lipid-gene signature exhibited good performance when distinguishing TB-only patients from non-TB controls without HIV (AUC of ROC curve and standard deviation from the 10-fold nested cross-validation = 0.766 ± 0.046) (**Figure 6A**). When differentiating between the TB-only group and non-TB controls with HIV, the classifier was not robustly against a random guess (the ROC crossed the diagonal line) (**Figure 6B**). The model showed an acceptable ability to differentiate the TB-only group from the OD (AUC = 0.679 ± 0.069) and OD-HIV (AUC = 0.690 ± 0.061) (**Figures 6C, D**). Additionally, the 10-lipid-gene signature showed excellent and good results in differentiating patients with TB from those with LTBI in GSE37250 (AUC = 0.832 ± 0.042) and in GSE107994 (AUC = 0.792 ± 0.067), respectively (**Figure 6E**, **Table 2**). Similarly, the 10-lipid-gene classifier distinguished the TB and LTBI-HIV groups with excellent performance (AUC = 0.871 ± 0.039) (**Figure 6F**). In the combined GSE42825/GSE42826/GSE42830 dataset, the 10-lipid-gene signature exhibited its best performance when distinguishing between patients with TB and healthy controls (AUC = 0.961 ± 0.027) (**Figure 6G**). Also, in this dataset, the 10-lipid-gene signature showed acceptable performance in distinguishing between TB and active SARC (AUC = 0.680 ± 0.079) and excellent performance in distinguishing between TB and non-active SARC (AUC = 0.877 ± 0.064) (**Figures 6H, I**). Other comparisons yielded acceptable accuracy (TB *versus* Cancer, AUC = 0.693 ± 0.076) or insignificant classification with a high variability (TB *versus* PNA, AUC = 0.760 ± 0.139) (**Figures 6J, K**).

**Table 2:** The performance of 10-lipid-gene signature for TB diagnosis in comparison with Long10, RISK6, and Sambarey10 signatures.

Dataset	Country	Comparison (number of patients)	AUC ± SD				FDR (Wilcoxon Rank Sum Test)		
			10-lipid-gene	Long10	RISK6	Sambarey10	Long10 *versus* 10-lipid-gene	RISK6 *versus* 10-lipid-gene	Sambarey10 *versus* 10-lipid-gene
E-MTAB-8290	South Africa	TB (37)/non-TB-non-HIV (100)	0.766 ± 0.046	0.851 ± 0.077	0.860 ± 0.068	0.877 ± 0.062	0.028	0.016	0.005
		TB (37)/non-TB-HIV (27)	0.742 ± 0.091	0.673 ± 0.072	0.748 ± 0.136	0.684 ± 0.16	0.148	1.000	0.590
		TB-HIV (17)/non-TB-non-HIV (100)	0.830 ± 0.111	0.949 ± 0.061	0.906 ± 0.083	0.925 ± 0.052	0.028	0.251	0.093
		TB-HIV (17)/non-TB-HIV (27)	0.762 ± 0.121	The SD was not sufficiently estimated	0.872 ± 0.115	0.790 ± 0.112	NA	0.157	0.622
		TB (37)/TB-HIV (17)	0.667 ± 0.142	0.591 ± 0.091	0.716 ± 0.130	0.596 ± 0.095	0.172	0.479	0.480
GSE37250*	Malawi, South Africa	TB (97)/OD (83)	0.679 ± 0.069	0.836 ± 0.045	0.798 ± 0.043	0.845 ± 0.055	4.90E-04	0.002	7.02E-04
		TB (97)/OD-HIV (92)	0.690 ± 0.061	0.812 ± 0.066	0.762 ± 0.052	0.907 ± 0.038	0.001	0.020	3.65E-04
		TB (97)/LTBI (83)	0.832 ± 0.042	0.955 ± 0.023	0.969 ± 0.018	0.942 ± 0.022	4.90E-04	6.39E-04	3.65E-04
		TB (97)/LTBI-HIV (84)	0.871 ± 0.039	0.939 ± 0.026	0.932 ± 0.033	0.954 ± 0.022	0.002	0.010	5.74E-04
		TB-HIV (98)/OD (83)	0.776 ± 0.062	0.912 ± 0.029	0.855 ± 0.048	0.917 ± 0.047	4.90E-04	0.002	6.80E-04
		TB-HIV (98)/OD-HIV (92)	0.601 ± 0.045	0.778 ± 0.044	0.861 ± 0.050	0.867 ± 0.036	4.90E-04	6.39E-04	3.65E-04
		TB-HIV (98)/LTBI (83)	0.833 ± 0.054	0.977 ± 0.010	0.975 ± 0.012	0.973 ± 0.011	4.90E-04	6.39E-04	3.65E-04
		TB-HIV (98)/LTBI-HIV (84)	0.813 ± 0.037	0.891 ± 0.036	0.866 ± 0.037	0.901 ± 0.066	0.001	0.006	0.008
		TB (97)/TB-HIV (98)	0.650 ± 0.057	0.780 ± 0.061	0.749 ± 0.041	0.837 ± 0.032	4.90E-04	0.002	3.65E-04
		LTBI (83)/OD (83)	0.814 ± 0.054	0.871 ± 0.054	0.869 ± 0.058	0.878 ± 0.039	0.025	0.048	0.008
		LTBI (83)/OD-HIV (92)	0.842 ± 0.050	0.924 ± 0.032	0.921 ± 0.024	0.947 ± 0.022	0.001	0.002	3.65E-04
		LTBI-HIV (84)/OD (83)	0.861 ± 0.060	0.913 ± 0.045	0.877 ± 0.057	0.905 ± 0.038	0.031	0.700	0.140
		LTBI-HIV (84)/OD-HIV (92)	0.868 ± 0.048	0.774 ± 0.039	0.875 ± 0.050	0.894 ± 0.034	0.001	0.791	0.257
		LTBI-HIV (84)/LTBI (83)	0.589 ± 0.072	0.873 ± 0.032	0.872 ± 0.039	0.873 ± 0.036	4.90E-04	6.39E-04	3.65E-04
GSE107991	United Kingdom	TB (21)/Control (12)	The SD was not sufficiently estimated	The SD was not sufficiently estimated	The SD was not sufficiently estimated	The SD was not sufficiently estimated	NA	NA	NA
		TB/LTBI (21)	The SD was not sufficiently estimated	The SD was not sufficiently estimated	0.850 ± 0.106	The SD was not sufficiently estimated	NA	NA	NA
		LTBI (21)/Control (12)	The SD was not sufficiently estimated	0.678 ± 0.128	0.756 ± 0.160	0.717 ± 0.149	NA	NA	NA
GSE107994	United Kingdom	TB (43)/Control (10)	The SD was not sufficiently estimated	The SD was not sufficiently estimated	The SD was not sufficiently estimated	The SD was not sufficiently estimated	NA	NA	NA
		TB (43)/LTBI (45)	0.792 ± 0.067	0.904 ± 0.066	0.910 ± 0.066	0.912 ± 0.053	0.006	0.006	0.004
		LTBI (45)/Control (10)	0.579 ± 0.091	0.697 ± 0.107	0.615 ± 0.088	0.636 ± 0.117	0.021	0.519	0.288
GSE101705	India	TB (28)/LTBI (16)	The SD was not sufficiently estimated	The SD was not sufficiently estimated	The SD was not sufficiently estimated	The SD was not sufficiently estimated	NA	NA	NA
GSE42825, GSE42826, GSE42830*¶#	United Kingdom, France	TB (35)/Control (113)	0.961 ± 0.027	The SD was not sufficiently estimated	The SD was not sufficiently estimated	The SD was not sufficiently estimated	NA	NA	NA
		TB (35)/Active SARC (39)	0.680 ± 0.079	0.803 ± 0.068	0.785 ± 0.076	0.859 ± 0.074	*0.004*	0.014	*9.95E-04*
		TB (35)/Non-active SARC (22)	0.877 ± 0.064	The SD was not sufficiently estimated	0.975 ± 0.024	The SD was not sufficiently estimated	NA	0.001	NA
		TB (35)/Cancer (16)	0.693 ± 0.076	The SD was not sufficiently estimated	0.887 ± 0.144	The SD was not sufficiently estimated	NA	0.018	NA
		TB (35)/PNA (14)	0.760 ± 0.139	The SD was not sufficiently estimated	0.930 ± 0.057	The SD was not sufficiently estimated	NA	0.013	NA

AUC, Area under the receiver operating characteristic curve; SD, standard deviation calculated from the 10-fold nested cross-validation; FDR, False Discovery Rate; TB, Tuberculosis (without HIV); HIV, Human Immunodeficiency Virus; TB-HIV, Tuberculosis with HIV; LTBI, Latent tuberculosis infection (without HIV); LTBI-HIV, Latent tuberculosis infection with HIV; OD, Other diseases (without HIV); OD-HIV, Other diseases with HIV; SARC, Sarcoidosis (without HIV); Cancer, Lung cancer (without HIV); PNA, Pneumonia (without HIV).

Italic value: raw P-value was used instead of FDR.

*The Long10 signature could not be fully retrieved from this dataset.

¶The 10-lipid-gene signature could not be fully retrieved from this dataset.

# The RISK6 signature could not be fully retrieved from this dataset.

NA, Not available.

The results from the comparison between TB-HIV and non-TB–non-HIV groups did not show strong evidence against a random guess (the ROC crossed the diagonal line) (**Figure 6L**). In contrast, the logistic regression classifier differentiated TB-HIV from non-TB-HIV with good accuracy (AUC = 0.762 ± 0.121) (**Figure 6M**). The performance of classifier in comparing the TB-HIV group with the OD (AUC = 0.776 ± 0.062), OD-HIV (AUC = 0.601 ± 0.045), LTBI (AUC = 0.833 ± 0.054), and LTBI-HIV (AUC = 0.813 ± 0.037) was comparable to that for the comparison of the TB-only group with these groups (**Figures 6N–Q**). Noticeably, the model could distinguish between TB and TB-HIV in GSE37250 with acceptable performance (AUC = 0.650 ± 0.057) (**Figure 6R**). Nevertheless, the results from the same comparison in E-MTAB-8290 were insignificant with a high variability (AUC = 0.667 ± 0.142) (**Table 2**). Additionally, the 10-lipid-gene signature exhibited an excellent ability to differentiate LTBI with OD (AUC = 0.814 ± 0.054) and OD-HIV (AUC = 0.842 ± 0.050) in the GSE37250 cohort (**Table 2**). Similar results were observed for the comparisons of LTBI-HIV with OD (AUC = 0.861 ± 0.060) and OD-HIV (AUC = 0.868 ± 0.048) (**Table 2**). The classifications between LTBI and healthy control (AUC = 0.579 ± 0.091) as well as LTBI-HIV and LTBI (AUC = 0.589 ± 0.072) were insignificant due to their high variability.

Where applicable, the diagnostic performance of the 10-lipid-gene signature was compared with that of other signatures (**Table 2**). In brief, the performance of the 10-lipid-gene signature was significantly poorer than that of the other signatures in most datasets. Nevertheless, the performance of the 10-lipid-gene signature was comparable to that of the RISK6 and Sambery10 signatures for certain comparisons in E-MTAB-8290, GSE37250, and GSE107994. They were TB with or without HIV *versus* non-TB-HIV in E-MTAB-8290, LTBI-HIV versus OD with or without HIV in GSE37250, and LTBI versus healthy control in GSE107994. Taken together, our results demonstrate the potential usefulness of the 10-lipid-gene signature for TB diagnosis in certain scenarios.

## 4 Discussion

The implementation of the WHO End TB strategy requires concerted efforts from the global scientific community to end the TB epidemic. A key focus area is the development of new tools for TB diagnosis, treatment monitoring, vaccine development, and therapeutic discovery. In recent years, host-based transcriptomic biosignature for TB diagnosis and treatment monitoring has been endorsed by scientific communities (13, 32, 36, 66–68). TB entails a spectrum of pathophysiological processes from the infection to the treatment completion stage (36), including inflammatory, interferon, immune, and T- and B-cell pathways (28, 69, 70). These pathophysiological events and molecular abnormalities can be evaluated by transcriptomics (70). Furthermore, although the interindividual variability of host responses is high, transcriptome-based signatures may display stable patterns during TB treatment, making them potential for treatment monitoring (71). Focusing on genes with clear patterns of changes during anti-TB treatment would help to discover relevant biomarkers, which together may form a robust predictive signature (72). In the present study, we evaluated lipid-related genes because previous studies have demonstrated the host immunological responses and lipidome alterations in TB and during anti-TB treatment (26, 27). Additionally, a knowledge-based and targeted approach derived from functional interpretation and mechanistic understanding may overcome the challenges of an entirely data-driven approach (16). These challenges include limited sample sizes, differences in the design of available data sets, the high-dimensional nature of transcriptomics data, and the lack of validation of particular signatures (9).

We employed a workflow that combined data-driven and knowledge-based approaches to investigate the expression of lipid-related genes during anti-TB treatment and its potential application for TB diagnosis in diverse clinical settings. A 10-lipid-gene signature showing clear changes during anti-TB treatment was established using time-course regression analysis. The potential usefulness of this signature for treatment monitoring was compared with that of three other signatures (*i.e.*, RISK6, Sambarey10, and our previously reported Long10 signature) in three cohorts using GSVA. GSVA provides a direct way for a head-to-head comparison between different signatures. Wang et al. found that scores for signatures obtained with gene set enrichment methods could differentiate between active TB and other clinical conditions with equivalent or better accuracy than could conventional methods (73). The GSVA scores for all four signatures differed significantly in investigated cohorts, perhaps because of the inclusion of genes playing a critical role in TB immune-signaling pathways. The 10-lipid-gene signature generally showed poor results when classifying different TB treatment states. However, its performance was not far behind RISK6 and Sambarey10 signatures. Only the Long10 signature exhibited acceptable performance in all investigated datasets. It is worth noting that, among all longitudinal validating datasets, the shortest time point was after one week of treatment (GSE89403), and the minimum sample size was 29 TB patients with samples collected from three time points for each patient (GSE181143 – subset from Brazil). Among 10-lipid-gene signature, some genes are found on immune cells, such as monocytes (e.g., *ARPC5*), neutrophils (e.g., *MBOAT1*, *MBOAT2*), lymphocytes (e.g., *ARPC5*), dendritic cells (e.g., *PLD4*) (65, 74–77). The expression level of these genes on immune cells as well as the frequency of cell source population could lead to difference of performance between the investigated datasets. Overall, the benchmark analysis results highlighted the role of lipid-related genes in TB pathophysiology. Nevertheless, the 10-lipid-gene signature has certain limitations and may not be the optimal choice for accurate monitoring of the anti-TB treatment response. Our results reinforced the usefulness of gene signatures related to the immune response for anti-TB treatment monitoring.

The usefulness of the 10-lipid-gene signature for the differentiation of active TB and non-TB counterparts was investigated in multiple clinical cohorts. In multiple comparisons, GSVA scores for the 10-lipid-gene signature were higher in the TB group than in other groups, with a remarkable ability to differentiate the TB and LTBI groups in all tested cohorts. These also indicated the association between the activation of lipid-related genes with TB disease. However, the performance of the 10-lipid-gene signature was generally not as excellent as other signatures because the expression of lipid-related genes changed only subtly. The caution should also be made since the OD group consisted of multiple respiratory diseases, which might introduce bias into the analysis (31). Consistent with the GSVA scores, the logistic regression classifier based on the 10-lipid-gene signature performed well in differentiating patients with TB from non-TB controls and those with LTBI (with or without HIV), and non-active SARC. The 10-lipid-gene signature had the best results for distinguishing TB from healthy controls. Noticeably, the good performance of the 10-lipid-gene signature in differentiating between TB and non-TB controls, healthy controls, LTBI, non-active SARC but not between TB and OD, active SARC, lung cancer, PNA indicates its limited capacity for TB differential diagnosis. The results are consistent with our previous investigations of lipid and lipid-related genes to diagnose active TB disease (26). Host lipids play a vital role in the immune response to TB infection. Our findings further confirm the role of lipid-related genes in the dysregulated host metabolism and immune signaling during TB activation relative to LTBI. Of note, the TB and TB-HIV groups could not be classified significantly across all cohorts, possibly due to heterogeneity among cohorts and relatively small sample sizes in the tested datasets. For instance, the status of antiretroviral therapy, which can alter the transcriptome of HIV patients (78), differs across cohorts and may partially contribute to variations in the performance of the signatures. Besides, similar shortcomings associated with small sample sizes for biomarker validation have been reported in other biomarker studies, such as RISK4 and RISK6 (36, 79). The 10-lipid-gene signature displayed unsatisfactory performance in differentiating patients with LTBI from healthy controls, concordant with the fact that metabolomes and lipidomes are similar between these groups (80). Overall, the 10-lipid-gene signature exhibited the potential to be used for further optimization of TB diagnosis.

At the current setup of biosignature, metabolism-centric biomarkers may not outperform other leading signatures. However, individual biomarkers reported in our work could be strong candidates to be considered when establishing a biosignature that takes into account the metabolic alterations during TB treatment. We provided proof-of-concept results regarding the potential of lipid biomarkers (27). These findings collectively demonstrate that metabolism-centric biomarkers could be a significant aspect to be explored further in addition to approaches targeting immunological processes.

The biological relevance of derived biosignature must be examined thoroughly due to its significance. The products encoded by the 10 candidate genes are involved in multiple immune processes (**Table 1**). For instance, subunit 5 of actin-related protein 2/3 complex, encoded by *ARPC5*, involves in the entry of *Mtb* into lung epithelial cells (81) as well as lymphocyte activation, adhesion, and migration, which are hallmarks of the TB pathophysiology (82–84). ACSL4 regulates ferroptosis by modulating the cellular lipidome (52). Furthermore, *ACSL4* was found to be overexpressed in anti-TB drug-induced liver injury, indicating ferroptosis induction during anti-TB treatment (85). *PLD4* is differentially expressed in patients with TB (27). Phospholipase D activation is associated closely with *Mtb* phagocytosis by macrophages (86). During the early stages of TB infection, *Mtb* inhibits phagosome maturation and acidification by various bacterial factors (87, 88). As the treatment progression with *Mtb* elimination, this inhibition is reduced. Additionally, the increase in the interferon-γ level during anti-TB treatment induces phagosome maturation in macrophages (89). The rise of phagocytosis could be associated with the elevation of *PLD4* gene expression during the TB treatment time course. Lysosomal acid lipase, encoded by *LIPA*, is involved in the maturation and function of immune cells via the regulation of FC and FFA levels (56). Interestingly, the rs1051338 and rs7922269 single-nucleotide polymorphisms of *LIPA* are associated with individual susceptibility to pulmonary TB (90). The CHMP2B protein is a subunit of the endosomal sorting complex required for transport III (ESCRT-III) (57). ESCRT-III is recruited and engaged with *Mtb* phagosomes, preventing *Mtb* release into the cytosol (91). *RAB5A* encodes a crucial small GTPase that regulates the fusion between bacteria-containing phagosomes (including *Mtb*) and cytoplasmic organelles (59), thereby influencing the ability of neutrophils to restrict pathogen spread (59, 60). Moreover, RAB5A is tightly involved in TB immune infiltration (92). The GABARAPL2 protein participates in the autophagy pathway an essential biological process that defends against intracellular microbes, including *Mtb* (61, 93). *Mtb*-dependent macrophage apoptosis requires phospholipase A2 group IVA, encoded by *PLA2G4A* (94). Phospholipase A2 group IVA is also responsible for the initial step in the arachidonic acid (AA) pathway, which involves the cleavage of AA from the sn-2 position of phospholipids in cell membranes (63). AA promotes the formation of eicosanoids, crucial inflammatory mediators (95). Interestingly, the two last components of our signature, MBOAT1 and MBOAT2, also regulate the free AA level through the arachidonate recycling process and relate to eicosanoids production (65, 95). AA-derived eicosanoids, including prostaglandins, leukotrienes, and lipoxins, can modulate the host response to *Mtb* infection (96, 97). Previous studies demonstrated the altered levels of eicosanoids in TB, TB with comorbid diabetes, and after TB treatment (98, 99). In general, the ten genes can be roughly categorized into three groups based on their associated immunological pathways. They are genes involved in apoptosis/phagocytosis/autophagy pathways (*CHMP2B*, *RAB5A*, *GABARAPL2*, *PLA2G4A*, *PLD4*), genes involved in AA/FFAs pathways (*PLA2G4A*, *MBOAT1*, *MBOAT2*, *LIPA*, *ACSL4*), and gene involves in lymphocyte migration (*ARPC5*). These findings suggest the existence of associations between lipid signaling and immune pathways.

This study has several limitations which should be assessed. Firstly, the biosignature was derived from a time-series analysis on a single cohort, which may limit the generalizability of the biosignature on diverse populations with heterogeneous backgrounds. We addressed the limitation by validating our signature in a cross-platform, multi-ethnic, multi-cohort scenario to demonstrate its applicability across diverse populations and settings. We further expanded the scope of our investigation beyond TB treatment monitoring to also include TB diagnostics, showcasing the flexibility of our signature. Furthermore, we conducted a head-to-head benchmarking analysis with other publicly available signatures to demonstrate the capacity of our signature. The second shortcoming is that we did not account for confounding factors during the time series analysis, which could potentially lead to false-positive signals. However, we mitigated this issue by adopting a targeted approach based on prior knowledge to minimize the number of false-positive findings. Thirdly, focusing on lipid-related genes, which exhibited subtle alteration between TB and its counterparts (26), might limit the robustness of the signature. However, finding a signature with excellent performance is an aim but not the primary goal of this study. Our study was conducted to demonstrate the potential of lipid-related gene markers in TB management and suggest the direction for subsequent studies. Moreover, the identification of certain genes as potential candidates might be attributed to their high correlation with the “true” markers. Indeed, the partial overlap between signatures is frequently observed. The 10-lipid-gene biosignature also intersects one gene (*MBOAT2*) with the 558-gene signature representing the TB treatment response of Bloom et al. (35). However, to the best of our knowledge, the remaining nine genes were reported for the first time in our study. This finding implicates that there is still ample room for further research in discovering metabolism-centric biomarkers, particularly lipid-related genes. Last but not least, exploring the integration of lipid-related genes with other signatures to enhance their performance should be pursued in future investigations.

In the present study, we developed a biosignature based on key lipid-related genes that can be used to assist the management of TB. Our findings emphasize the crucial role of lipid metabolism in TB pathophysiology and treatment response. Additionally, the lipid-related genes have been implicated in the host immune response, highlighting the significant association between lipid metabolism and the immune system in TB. This association presents a promising target for the development of novel TB diagnostic and treatment monitoring strategies. It should be explored further to enhance our understanding and improve TB management.

## Data availability statement

The original contributions presented in the study are included in the article/**Supplementary Material**. Further inquiries can be directed to the corresponding authors.

## Author contributions

Conceptualization, SP, NL, DK, and J-GS; Investigation, NP and NL; Formal Analysis, NP, SP, NL, and NT; Writing – Original draft, NP, NA, NL, DK, and J-GS; Writing – Review & Editing, NP, NT, NA, NY, YL, HT, K-ML, SA, Y-SC, SP, NL, DK, and J-GS; Visualization, NP, NT, and NY, Data curation, NP, NT, NA, and NY; Methodology, NP, NT, NA, NY, YL, HT, K-ML, SA, Y-SC, SP, NL, DK, and J-GS; Validation, NP, NT, NA, NY, YL, HT, K-ML, SA, Y-SC, SP, NL, DK, and J-GS; Supervision, SP, NL, DK, and J-GS; Resources, J-GS; Funding acquisition, J-GS. All authors contributed to the article and approved the submitted version.
