# Supplementary material for: Alterations of lipid-related genes during anti-tuberculosis treatment: insights into host immune responses and potential transcriptional biomarkers
Source: Front Immunol. 2023 Oct 31;14:1210372. doi: 10.3389/fimmu.2023.1210372 (PMC10644770; doi:10.3389/fimmu.2023.1210372)
Supplement: Supplementary file 1 [file DataSheet_1.docx]

Alterations of Lipid-Related Genes during Anti-Tuberculosis Treatment: Insights into Host Immune Responses and Potential Transcriptional Biomarkers

**Nguyen Ky Phat^1,2^, Nguyen Tran Nam Tien^1,2^, Nguyen Ky Anh^1,2^, Nguyen Thi Hai Yen^1,2^, Yoon Ah Lee^3^, Hoang Kim Tu Trinh^4^,** **Kieu-Minh Le^4^, Sangzin Ahn^1,2^, Yong-Soon Cho^1,2^, Seongoh Park^3,5^, Dong-Hyun Kim^1^, Nguyen Phuoc Long^1,2**^, Jae-Gook Shin^1,2*^**

^1^Department of Pharmacology and PharmacoGenomics Research Center, Inje University College of Medicine, Busan, Republic of Korea

^2^Center for Personalized Precision Medicine of Tuberculosis, Inje University College of Medicine, Busan, Republic of Korea

^3^School of Mathematics, Statistics and Data Science, Sungshin Women’s University, Seoul, Republic of Korea

^4^Center for Molecular Biomedicine, University of Medicine and Pharmacy at Ho Chi Minh, Ho Chi Minh City, Vietnam

^5^Data Science Center, Sungshin Women’s University, Seoul, Republic of Korea

*** Correspondence:**Jae-Gook Shin, MD, PhD
[phshinjg@inje.ac.kr](mailto:phshinjg@inje.ac.kr)

**** Correspondence:**Nguyen Phuoc Long, MD, PhD
[phuoclong@inje.ac.kr](mailto:phuoclong@inje.ac.kr)


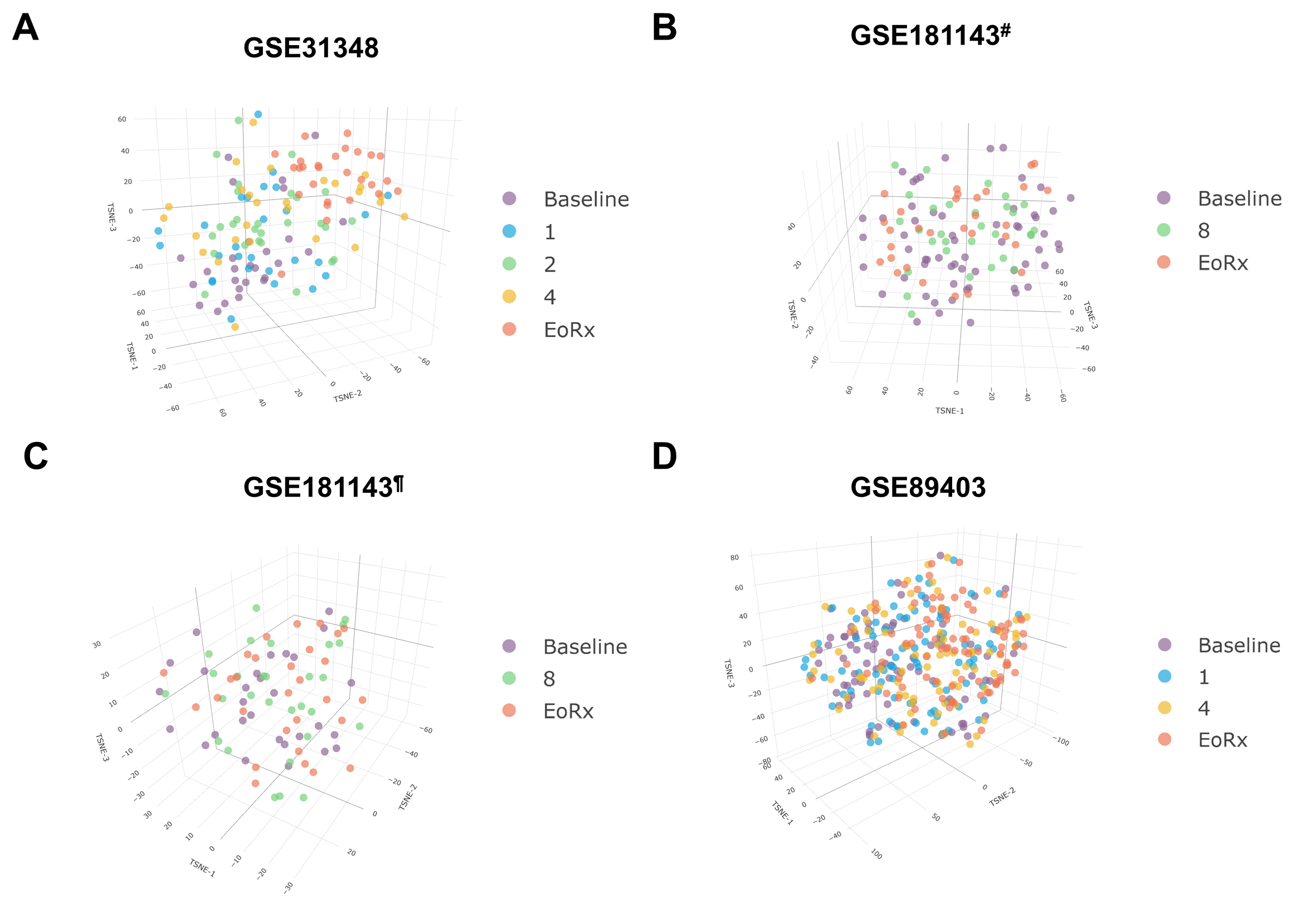


**Supplementary Figure 1.** Scores plots of t-distributed stochastic neighbor embedding represent the transcriptome profiles for 162 lipid-related genes during the TB treatment. (A) GSE31348. (B) GSE181143, cohort from India. (C) GSE181143, cohort from Brazil. (D) GSE89403. Abbreviations and remarks: 1, after one week; 2, after two weeks; 4, after four weeks; 8, after eight weeks; EoRx, treatment completion; #, subset from India of GSE181143 dataset; ¶, subset from Brazil of GSE181143 dataset.

**Supplementary Table 1**. Description of the published dataset used in this study.

| **Dataset** | | | GSE31348 | GSE89403 | GSE181143 | E-MTAB-8290 | GSE37250 | GSE107991 | GSE107994 | GSE101705 | GSE42825 | GSE42826 | GSE42830 |
| --- | --- | --- | --- | --- | --- | --- | --- | --- | --- | --- | --- | --- | --- |
| **PMID** | | | [22872737](https://www.ncbi.nlm.nih.gov/pubmed/22872737) | [29050771](https://www.ncbi.nlm.nih.gov/pubmed/29050771) | [37173394](https://www.ncbi.nlm.nih.gov/pubmed/37173394) | 32178775 | [24167453](https://www.ncbi.nlm.nih.gov/pubmed/24167453) | [29921861](https://www.ncbi.nlm.nih.gov/pubmed/29921861) | [29921861](https://www.ncbi.nlm.nih.gov/pubmed/29921861) | 29559120 | [23940611](https://www.ncbi.nlm.nih.gov/pubmed/23940611) | [23940611](https://www.ncbi.nlm.nih.gov/pubmed/23940611) | [23940611](https://www.ncbi.nlm.nih.gov/pubmed/23940611) |
| **Published year** | | | 2012 | 2017 | 2021 | 2020 | 2013 | 2018 | 2018 | 2018 | 2013 | 2013 | 2013 |
| **Region** | | | South Africa | South Africa | India, Brazil | South Africa | South Africa, Malawi | United Kingdom | United Kingdom | India | United Kingdom, France | United Kingdom, France | United Kingdom, France |
| **Platform** | | | Affymetrix Human Genome U133 Plus 2.0 Array | Illumina HiSeq 2000 | HiSeq X Ten | Illumina NextSeq 500 | Illumina HumanHT-12 V4.0 expression beadchip | Illumina HiSeq 4000 | Illumina HiSeq 4000 | Illumina NextSeq 500 | Illumina HumanHT-12 V4.0 expression beadchip | Illumina HumanHT-12 V4.0 expression beadchip | Illumina HumanHT-12 V4.0 expression beadchip |
| **Repeated Measurement Study Design** | | | Yes | Yes | Yes | No | No | No | No | No | No | No | No |
| **Number of time point** | | | 5 | 4 | 3 |  |  |  |  |  |  |  |  |
| **Sample size** | TB | TB-HIV | 27 | 90 | 89 | 17 | 98 | 21 | 43 | 28 | 8 | 11 | 16 |
|  |  | TB-non-HIV |  |  |  | 37 | 97 |  |  |  |  |  |  |
|  | non-TB | non-TB-HIV |  |  |  | 27 |  |  |  |  |  |  |  |
|  |  | non-TB-non-HIV |  |  |  | 100 |  |  |  |  |  |  |  |
|  | LTBI | LTBI-HIV |  |  |  |  | 84 | 21 | 45 | 16 |  |  |  |
|  |  | LTBI |  |  |  |  | 83 |  |  |  |  |  |  |
|  | OD | OD-HIV |  |  |  |  | 92 |  |  |  |  |  |  |
|  |  | OD |  |  |  |  | 83 |  |  |  |  |  |  |
|  | Active SARC | |  |  |  |  |  |  |  |  | 6 | 16 | 17 |
|  | Non-active SARC | |  |  |  |  |  |  |  |  | 5 | 9 | 8 |
|  | Cancer | |  |  |  |  |  |  |  |  |  | 8 | 8 |
|  | PNA | |  |  |  |  |  |  |  |  |  | 6 | 8 |
|  | Healthy Control | |  |  |  |  |  | 12 | 10 |  | 23 | 52 | 38 |
| **TB**: Tuberculosis (without HIV)  **HIV**: Human Immunodeficiency Virus  **TB-HIV**: Tuberculosis with HIV  **LTBI**: Latent tuberculosis infection (without HIV)  **LTBI-HIV**: Latent tuberculosis infection with HIV  **OD**: Other diseases (without HIV)  **OD-HIV**: Other diseases with HIV  **SARC**: Sarcoidosis (without HIV)  **Cancer**: Lung cancer (without HIV)  **PNA**: Pneumonia (without HIV) | | | | | | | | | | | | | |

**Supplementary Table 2**. List of 162 lipid-related genes that are potential biomarker candidates for treatment monitoring.

| **EntrezID** | **Gene Symbol** | **Description** |
| --- | --- | --- |
| 356 | *FASLG* | Fas ligand |
| 3551 | *IKBKB* | inhibitor of nuclear factor kappa B kinase subunit beta |
| 5879 | *RAC1* | Rac family small GTPase 1 |
| 7099 | *TLR4* | toll like receptor 4 |
| 998 | *CDC42* | cell division cycle 42 |
| 5602 | *MAPK10* | mitogen-activated protein kinase 10 |
| 5599 | *MAPK8* | mitogen-activated protein kinase 8 |
| 5601 | *MAPK9* | mitogen-activated protein kinase 9 |
| 5337 | *PLD1* | phospholipase D1 |
| 5338 | *PLD2* | phospholipase D2 |
| 135 | *ADORA2A* | adenosine A2a receptor |
| 136 | *ADORA2B* | adenosine A2b receptor |
| 10390 | *CEPT1* | choline/ethanolamine phosphotransferase 1 |
| 56994 | *CHPT1* | choline phosphotransferase 1 |
| 8681 | *JMJD7-PLA2G4B* | JMJD7-PLA2G4B readthrough |
| 79888 | *LPCAT1* | lysophosphatidylcholine acyltransferase 1 |
| 54947 | *LPCAT2* | lysophosphatidylcholine acyltransferase 2 |
| 254531 | *LPCAT4* | lysophosphatidylcholine acyltransferase 4 |
| 5048 | *PAFAH1B1* | platelet activating factor acetylhydrolase 1b regulatory subunit 1 |
| 5049 | *PAFAH1B2* | platelet activating factor acetylhydrolase 1b catalytic subunit 2 |
| 5050 | *PAFAH1B3* | platelet activating factor acetylhydrolase 1b catalytic subunit 3 |
| 8399 | *PLA2G10* | phospholipase A2 group X |
| 81579 | *PLA2G12A* | phospholipase A2 group XIIA |
| 84647 | *PLA2G12B* | phospholipase A2 group XIIB |
| 5319 | *PLA2G1B* | phospholipase A2 group IB |
| 5320 | *PLA2G2A* | phospholipase A2 group IIA |
| 391013 | *PLA2G2C* | phospholipase A2 group IIC |
| 26279 | *PLA2G2D* | phospholipase A2 group IID |
| 30814 | *PLA2G2E* | phospholipase A2 group IIE |
| 64600 | *PLA2G2F* | phospholipase A2 group IIF |
| 50487 | *PLA2G3* | phospholipase A2 group III |
| 5321 | *PLA2G4A* | phospholipase A2 group IVA |
| 100137049 | *PLA2G4B* | phospholipase A2 group IVB |
| 8605 | *PLA2G4C* | phospholipase A2 group IVC |
| 283748 | *PLA2G4D* | phospholipase A2 group IVD |
| 123745 | *PLA2G4E* | phospholipase A2 group IVE |
| 255189 | *PLA2G4F* | phospholipase A2 group IVF |
| 5322 | *PLA2G5* | phospholipase A2 group V |
| 8398 | *PLA2G6* | phospholipase A2 group VI |
| 11145 | *PLAAT3* | phospholipase A and acyltransferase 3 |
| 151056 | *PLB1* | phospholipase B1 |
| 23646 | *PLD3* | phospholipase D family member 3 |
| 122618 | *PLD4* | phospholipase D family member 4 |
| 85465 | *SELENOI* | selenoprotein I |
| 255043 | *TMEM86B* | transmembrane protein 86B |
| 335 | *APOA1* | apolipoprotein A1 |
| 337 | *APOA4* | apolipoprotein A4 |
| 338 | *APOB* | apolipoprotein B |
| 948 | *CD36* | CD36 molecule |
| 1056 | *CEL* | carboxyl ester lipase |
| 1208 | *CLPS* | colipase |
| 8694 | *DGAT1* | diacylglycerol O-acyltransferase 1 |
| 84649 | *DGAT2* | diacylglycerol O-acyltransferase 2 |
| 2168 | *FABP1* | fatty acid binding protein 1 |
| 2169 | *FABP2* | fatty acid binding protein 2 |
| 8513 | *LIPF* | lipase F, gastric type |
| 346606 | *MOGAT3* | monoacylglycerol O-acyltransferase 3 |
| 4547 | *MTTP* | microsomal triglyceride transfer protein |
| 5406 | *PNLIP* | pancreatic lipase |
| 5407 | *PNLIPRP1* | pancreatic lipase related protein 1 |
| 5408 | *PNLIPRP2* | pancreatic lipase related protein 2 (gene/pseudogene) |
| 949 | *SCARB1* | scavenger receptor class B member 1 |
| 160 | *AP2A1* | adaptor related protein complex 2 subunit alpha 1 |
| 161 | *AP2A2* | adaptor related protein complex 2 subunit alpha 2 |
| 163 | *AP2B1* | adaptor related protein complex 2 subunit beta 1 |
| 1173 | *AP2M1* | adaptor related protein complex 2 subunit mu 1 |
| 1175 | *AP2S1* | adaptor related protein complex 2 subunit sigma 1 |
| 1211 | *CLTA* | clathrin light chain A |
| 1212 | *CLTB* | clathrin light chain B |
| 1213 | *CLTC* | clathrin heavy chain |
| 8218 | *CLTCL1* | clathrin heavy chain like 1 |
| 1759 | *DNM1* | dynamin 1 |
| 1785 | *DNM2* | dynamin 2 |
| 26052 | *DNM3* | dynamin 3 |
| 10097 | *ACTR2* | actin related protein 2 |
| 10096 | *ACTR3* | actin related protein 3 |
| 273 | *AMPH* | amphiphysin |
| 10552 | *ARPC1A* | actin related protein 2/3 complex subunit 1A |
| 10109 | *ARPC2* | actin related protein 2/3 complex subunit 2 |
| 10094 | *ARPC3* | actin related protein 2/3 complex subunit 3 |
| 10093 | *ARPC4* | actin related protein 2/3 complex subunit 4 |
| 10092 | *ARPC5* | actin related protein 2/3 complex subunit 5 |
| 274 | *BIN1* | bridging integrator 1 |
| 27243 | *CHMP2A* | charged multivesicular body protein 2A |
| 25978 | *CHMP2B* | charged multivesicular body protein 2B |
| 51652 | *CHMP3* | charged multivesicular body protein 3 |
| 29082 | *CHMP4A* | charged multivesicular body protein 4A |
| 128866 | *CHMP4B* | charged multivesicular body protein 4B |
| 92421 | *CHMP4C* | charged multivesicular body protein 4C |
| 79643 | *CHMP6* | charged multivesicular body protein 6 |
| 91782 | *CHMP7* | charged multivesicular body protein 7 |
| 1601 | *DAB2* | DAB adaptor protein 2 |
| 9829 | *DNAJC6* | DnaJ heat shock protein family (Hsp40) member C6 |
| 2060 | *EPS15* | epidermal growth factor receptor pathway substrate 15 |
| 3312 | *HSPA8* | heat shock protein family A (Hsp70) member 8 |
| 3949 | *LDLR* | low density lipoprotein receptor |
| 26119 | *LDLRAP1* | low density lipoprotein receptor adaptor protein 1 |
| 5868 | *RAB5A* | RAB5A, member RAS oncogene family |
| 5869 | *RAB5B* | RAB5B, member RAS oncogene family |
| 5878 | *RAB5C* | RAB5C, member RAS oncogene family |
| 6455 | *SH3GL1* | SH3 domain containing GRB2 like 1, endophilin A2 |
| 6456 | *SH3GL2* | SH3 domain containing GRB2 like 2, endophilin A1 |
| 6457 | *SH3GL3* | SH3 domain containing GRB2 like 3, endophilin A3 |
| 8976 | *WASL* | WASP like actin nucleation promoting factor |
| 259230 | *SGMS1* | sphingomyelin synthase 1 |
| 166929 | *SGMS2* | sphingomyelin synthase 2 |
| 7124 | *TNF* | tumor necrosis factor |
| 9140 | *ATG12* | autophagy related 12 |
| 55054 | *ATG16L1* | autophagy related 16 like 1 |
| 115201 | *ATG4A* | autophagy related 4A cysteine peptidase |
| 23192 | *ATG4B* | autophagy related 4B cysteine peptidase |
| 84938 | *ATG4C* | autophagy related 4C cysteine peptidase |
| 84971 | *ATG4D* | autophagy related 4D cysteine peptidase |
| 9474 | *ATG5* | autophagy related 5 |
| 8678 | *BECN1* | beclin 1 |
| 11337 | *GABARAP* | GABA type A receptor-associated protein |
| 23710 | *GABARAPL1* | GABA type A receptor associated protein like 1 |
| 11345 | *GABARAPL2* | GABA type A receptor associated protein like 2 |
| 5289 | *PIK3C3* | phosphatidylinositol 3-kinase catalytic subunit type 3 |
| 30849 | *PIK3R4* | phosphoinositide-3-kinase regulatory subunit 4 |
| 26100 | *WIPI2* | WD repeat domain, phosphoinositide interacting 2 |
| 56894 | *AGPAT3* | 1-acylglycerol-3-phosphate O-acyltransferase 3 |
| 3931 | *LCAT* | lecithin-cholesterol acyltransferase |
| 10162 | *LPCAT3* | lysophosphatidylcholine acyltransferase 3 |
| 154141 | *MBOAT1* | membrane bound O-acyltransferase domain containing 1 |
| 129642 | *MBOAT2* | membrane bound O-acyltransferase domain containing 2 |
| 10400 | *PEMT* | phosphatidylethanolamine N-methyltransferase |
| 23761 | *PISD* | phosphatidylserine decarboxylase |
| 9791 | *PTDSS1* | phosphatidylserine synthase 1 |
| 81490 | *PTDSS2* | phosphatidylserine synthase 2 |
| 6901 | *TAFAZZIN* | tafazzin, phospholipid-lysophospholipid transacylase |
| 2268 | *FGR* | FGR proto-oncogene, Src family tyrosine kinase |
| 336 | *APOA2* | apolipoprotein A2 |
| 341 | *APOC1* | apolipoprotein C1 |
| 344 | *APOC2* | apolipoprotein C2 |
| 345 | *APOC3* | apolipoprotein C3 |
| 348 | *APOE* | apolipoprotein E |
| 1071 | *CETP* | cholesteryl ester transfer protein |
| 3988 | *LIPA* | lipase A, lysosomal acid type |
| 3990 | *LIPC* | lipase C, hepatic type |
| 9388 | *LIPG* | lipase G, endothelial type |
| 4018 | *LPA* | lipoprotein(a) |
| 4023 | *LPL* | lipoprotein lipase |
| 4035 | *LRP1* | LDL receptor related protein 1 |
| 4036 | *LRP2* | LDL receptor related protein 2 |
| 5360 | *PLTP* | phospholipid transfer protein |
| 4952 | *OCRL* | OCRL inositol polyphosphate-5-phosphatase |
| 5286 | *PIK3C2A* | phosphatidylinositol-4-phosphate 3-kinase catalytic subunit type 2 alpha |
| 8867 | *SYNJ1* | synaptojanin 1 |
| 8871 | *SYNJ2* | synaptojanin 2 |
| 23205 | *ACSBG1* | acyl-CoA synthetase bubblegum family member 1 |
| 81616 | *ACSBG2* | acyl-CoA synthetase bubblegum family member 2 |
| 2180 | *ACSL1* | acyl-CoA synthetase long chain family member 1 |
| 2181 | *ACSL3* | acyl-CoA synthetase long chain family member 3 |
| 2182 | *ACSL4* | acyl-CoA synthetase long chain family member 4 |
| 51703 | *ACSL5* | acyl-CoA synthetase long chain family member 5 |
| 23305 | *ACSL6* | acyl-CoA synthetase long chain family member 6 |
| 116519 | *APOA5* | apolipoprotein A5 |
| 2167 | *FABP4* | fatty acid binding protein 4 |
| 4973 | *OLR1* | oxidized low density lipoprotein receptor 1 |
| 5465 | *PPARA* | peroxisome proliferator activated receptor alpha |
| 7350 | *UCP1* | uncoupling protein 1 |

**Supplementary Table 3.** Results of Friedman test and *post hoc* two-sided paired Wilcoxon signed rank test to compare the GSVA score between any two time points for 162 lipid-related genes in GSE31348, GSE89403, and GSE181143 datasets.

| **Dataset** | **Country** | **Comparison** | **P-value** | **FDR** |
| --- | --- | --- | --- | --- |
|  |  |  | Friedman Test | Two-sided Paired Wilcoxon Rank Sum Test |
| GSE31348 | South Africa | Baseline/Week 1 | 2.31E-11 | 4.84E-04 |
|  |  | Baseline/Week 2 |  | 2.00E-05 |
|  |  | Baseline/Week 4 |  | 4.84E-04 |
|  |  | Baseline/Week 26 |  | 2.98E-07 |
|  |  | Week 1/Week 2 |  | 0.696 |
|  |  | Week 1/Week 4 |  | 0.572 |
|  |  | Week 1/Week 26 |  | 2.00E-05 |
|  |  | Week 2/Week 4 |  | 0.572 |
|  |  | Week 2/Week 26 |  | 1.89E-05 |
|  |  | Week 4/Week 26 |  | 9.50E-05 |
| GSE89403 | South Africa | Baseline/Week 1 | 8.02E-07 | 4.88E-04 |
|  |  | Baseline/Week 4 |  | 3.36E-04 |
|  |  | Baseline/Week 24 |  | 2.35E-07 |
|  |  | Week 1/Week 4 |  | 0.484 |
|  |  | Week 1/Week 24 |  | 0.011 |
|  |  | Week 4/Week 24 |  | 0.006 |
| GSE181143 | India | Baseline/Week 8 | 2.66E-04 | 1.68E-04 |
|  |  | Baseline/Week 24 |  | 5.70E-04 |
|  |  | Week 8/Week 24 |  | 0.641 |
| GSE181143 | Brazil | Baseline/Week 8 | 0.013 | 0.033 |
|  |  | Baseline/Week 24 |  | 0.033 |
|  |  | Week 8/Week 24 |  | 0.966 |
| **FDR**: False Discovery Rate | | | | |

**Supplementary Table 4.** Results of Kruskal-Wallis test and *post hoc* two-sided Wilcoxon rank sum test to compare the GSVA score between any two subject groups for 162 lipid-related genes in E-MTAB-8290, GSE37250, GSE107991, GSE107994, GSE101705, GSE42825, GSE42826, GSE42830 datasets.

| **Dataset** | **Comparison** | **P-value** | **FDR** |
| --- | --- | --- | --- |
|  |  | Kruskal-Wallis Test | Two-sided Wilcoxon Rank Sum Test |
| E-MTAB-8290 | TB/non-TB-non-HIV | 0.001 | 0.012 |
|  | TB/non-TB-HIV |  | 0.200 |
|  | TB-HIV/non-TB-non-HIV |  | 0.012 |
|  | TB-HIV/non-TB-HIV |  | 0.112 |
|  | TB/TB-HIV |  | 0.427 |
|  | non-TB-HIV/non-TB-non-HIV |  | 0.209 |
| GSE37250 | TB/OD | 1.03E-21 | 0.160 |
|  | TB/OD-HIV |  | 0.793 |
|  | TB/LTBI |  | 1.84E-12 |
|  | TB/LTBI-HIV |  | 1.77E-11 |
|  | TB-HIV/OD |  | 0.003 |
|  | TB-HIV/OD-HIV |  | 0.248 |
|  | TB-HIV/LTBI |  | 1.74E-06 |
|  | TB-HIV/LTBI-HIV |  | 5.66E-06 |
|  | TB/TB-HIV |  | 0.077 |
|  | LTBI/OD |  | 1.84E-12 |
|  | LTBI/OD-HIV |  | 5.01E-08 |
|  | LTBI-HIV/OD |  | 1.77E-11 |
|  | LTBI-HIV/OD-HIV |  | 1.92E-07 |
|  | LTBI-HIV/LTBI |  | 0.927 |
|  | OD/OD-HIV |  | 0.158 |
| GSE107991 | TB/Control | 3.75E-04 | 0.001 |
|  | TB/LTBI |  | 9.24E-04 |
|  | LTBI/Control |  | 0.839 |
| GSE107994 | TB/Control | 6.38E-05 | 5.37E-04 |
|  | TB/LTBI |  | 9.54E-04 |
|  | LTBI/Control |  | 0.038 |
| GSE101705 | TB/LTBI | NA | 0.077 |
| GSE42825, GSE42826, GSE42830 | TB/Control | 3.42E-17 | 4.22E-10 |
|  | TB/Active SARC |  | 0.534 |
|  | TB/Non-active SARC |  | 0.013 |
|  | TB/Cancer |  | 0.680 |
|  | TB/PNA |  | 0.351 |
|  | Active SARC/Control |  | 7.15E-09 |
|  | Active SARC/Non-active SARC |  | 0.077 |
|  | Active SARC/Cancer |  | 0.351 |
|  | Active SARC/PNA |  | 0.077 |
|  | Non-active SARC/Control |  | 0.013 |
|  | Non-active SARC/Cancer |  | 0.036 |
|  | Non-active SARC/PNA |  | 0.013 |
|  | Cancer/Control |  | 7.50E-06 |
|  | Cancer/PNA |  | 0.534 |
|  | PNA/Control |  | 7.50E-06 |
| **FDR**: False Discovery Rate  **TB:** Tuberculosis (without HIV)  **HIV:** Human Immunodeficiency Virus  **TB-HIV:** Tuberculosis with HIV  **LTBI:** Latent tuberculosis infection (without HIV)  **LTBI-HIV**: Latent tuberculosis infection with HIV  **OD:** Other diseases (without HIV)  **OD-HIV:** Other diseases with HIV  **SARC:** Sarcoidosis (without HIV)  **Cancer:** Lung cancer (without HIV)  **PNA:** Pneumonia (without HIV) | | | |

**Supplementary Table 5**. Results of Friedman test and *post hoc* two-sided paired Wilcoxon signed rank test to compare the GSVA score between any two time points for 10-lipid-gene signature in GSE31348.

| **Comparison** | **P-value** | **FDR** |
| --- | --- | --- |
|  | Friedman Test | Two-sided Paired Wilcoxon Rank Sum Test |
| Baseline/Week 1 | 8.09E-13 | 0.034 |
| Baseline/Week 2 |  | 0.069 |
| Baseline/Week 4 |  | 6.04E-05 |
| Baseline/Week 26 |  | 4.97E-08 |
| Week 1/Week 2 |  | 0.239 |
| Week 1/Week 4 |  | 0.157 |
| Week 1/Week 26 |  | 4.97E-08 |
| Week 2/Week 4 |  | 0.008 |
| Week 2/Week 26 |  | 4.97E-08 |
| Week 4/Week 26 |  | 1.12E-07 |
| **FDR**: False Discovery Rate | | |

**Supplementary Table 6**. Results of Friedman test and *post hoc* two-sided paired Wilcoxon signed rank test to compare the GSVA score between any two time points for 10-lipid-gene, Long10, RISK6, and Samberey10 signatures in GSE89403, and GSE181143 datasets.

| **Dataset** | **Country** | **Comparison** | **10-lipid-gene** | | **Long10** | | **RISK6** | | **Samberey10** | |
| --- | --- | --- | --- | --- | --- | --- | --- | --- | --- | --- |
|  |  |  | P-value | FDR | P-value | FDR | P-value | FDR | P-value | FDR |
|  |  |  | Friedman Test | Two-sided Paired Wilcoxon Rank Sum Test | Friedman Test | Two-sided Paired Wilcoxon Rank Sum Test | Friedman Test | Two-sided Paired Wilcoxon Rank Sum Test | Friedman Test | Two-sided Paired Wilcoxon Rank Sum Test |
| GSE89403 | South Africa | Baseline/Week 1 | 5.98E-06 | 6.02E-07 | 9.56E-25 | 2.21E-11 | 5.60E-19 | 5.10E-08 | 2.77E-25 | 5.48E-11 |
|  |  | Baseline/Week 4 |  | 1.13E-05 |  | 3.90E-12 |  | 7.12E-07 |  | 1.89E-11 |
|  |  | Baseline/Week 24 |  | 1.23E-07 |  | 8.88E-12 |  | 7.34E-11 |  | 3.48E-12 |
|  |  | Week 1/Week 4 |  | 0.991 |  | 0.086 |  | 0.766 |  | 0.011 |
|  |  | Week 1/Week 24 |  | 0.099 |  | 8.97E-07 |  | 1.11E-05 |  | 7.68E-08 |
|  |  | Week 4/Week 24 |  | 0.013 |  | 2.22E-06 |  | 1.44E-05 |  | 1.30E-06 |
| GSE181143 | India | Baseline/Week 8 | 0.032 | 0.030 | 1.68E-07 | 1.22E-04 | 0.004 | 0.452 | 1.97E-05 | 9.39E-04 |
|  |  | Baseline/Week 24 |  | 0.001 |  | 5.59E-07 |  | 0.005 |  | 1.23E-06 |
|  |  | Week 8/Week 24 |  | 0.452 |  | 0.008 |  | 0.005 |  | 0.024 |
| GSE181143 | Brazil | Baseline/Week 8 | 0.013 | 0.014 | 2.53E-05 | 0.006 | 0.006 | 0.041 | 3.11E-05 | 0.005 |
|  |  | Baseline/Week 24 |  | 0.010 |  | 1.67E-04 |  | 9.68E-04 |  | 1.67E-04 |
|  |  | Week 8/Week 24 |  | 0.782 |  | 0.005 |  | 0.056 |  | 0.041 |
| **FDR**: False Discovery Rate | | | | | | | | | | |

**Supplementary Table 7**. Results of Kruskal-Wallis test and *post hoc* two-sided Wilcoxon rank sum test to compare the GSVA score between any two subject groups for 10-lipid-gene, Long10, RISK6, and Samberey10 signatures in E-MTAB-8290, GSE107991, GSE107994, GSE37250, GSE42825, GSE42826, and GSE42830 datasets.

| **Dataset** | **Comparison** | **10-lipid-gene** | | **Long10** | | **RISK6** | | **Sambarey10** | |
| --- | --- | --- | --- | --- | --- | --- | --- | --- | --- |
|  |  | P-value | FDR | P-value | FDR | P-value | False Discovery Rate | P-value | FDR |
|  |  | Kruskal-Wallis  Test | Two-sided Wilcoxon Rank Sum Test | Kruskal-Wallis  Test | Two-sided Wilcoxon Rank Sum Test | Kruskal-Wallis  Test | Two-sided Wilcoxon Rank Sum Test | Kruskal-Wallis  Test | Two-sided Wilcoxon Rank Sum Test |
| E-MTAB-8290 | TB/non-TB-non-HIV | 0.002 | 0.012 | 8.02E-10 | 2.11E-06 | 5.24E-10 | 2.76E-07 | 2.05E-09 | 2.11E-06 |
|  | TB/non-TB-HIV |  | 0.047 |  | 0.007 |  | 0.005 |  | 0.058 |
|  | TB-HIV/non-TB-non-HIV |  | 0.009 |  | 2.11E-06 |  | 1.36E-05 |  | 2.11E-06 |
|  | TB-HIV/non-TB-HIV |  | 0.034 |  | 4.25E-04 |  | 0.017 |  | 0.005 |
|  | TB/TB-HIV |  | 0.605 |  | 0.513 |  | 0.831 |  | 0.233 |
|  | non-TB-HIV/non-TB-non-HIV |  | 0.914 |  | 0.186 |  | 0.059 |  | 0.047 |
| GSE37250 | TB/OD | 1.73E-26 | 0.075 | 1.37E-49 | 7.78E-07 | 2.97E-26 | 1.69E-08 | 2.59E-47 | 4.00E-04 |
|  | TB/OD-HIV |  | 0.971 |  | 2.47E-06 |  | 2.17E-05 |  | 0.664 |
|  | TB/LTBI |  | 9.41E-11 |  | 8.22E-25 |  | 2.50E-12 |  | 6.06E-24 |
|  | TB/LTBI-HIV |  | 4.65E-12 |  | 1.23E-17 |  | 0.032 |  | 1.28E-16 |
|  | TB-HIV/OD |  | 0.027 |  | 2.47E-06 |  | 5.44E-13 |  | 1.61E-06 |
|  | TB-HIV/OD-HIV |  | 0.609 |  | 7.80E-06 |  | 3.73E-10 |  | 0.033 |
|  | TB-HIV/LTBI |  | 2.11E-08 |  | 9.39E-25 |  | 5.60E-17 |  | 6.06E-24 |
|  | TB-HIV/LTBI-HIV |  | 9.81E-10 |  | 6.84E-17 |  | 6.44E-05 |  | 3.27E-17 |
|  | TB/TB-HIV |  | 0.628 |  | 0.956 |  | 0.139 |  | 0.099 |
|  | LTBI/OD |  | 7.28E-14 |  | 3.66E-16 |  | 0.079 |  | 2.42E-16 |
|  | LTBI/OD-HIV |  | 8.03E-11 |  | 8.66E-22 |  | 2.04E-05 |  | 5.90E-22 |
|  | LTBI-HIV/OD |  | 4.10E-15 |  | 3.05E-05 |  | 1.47E-05 |  | 6.20E-08 |
|  | LTBI-HIV/OD-HIV |  | 1.07E-12 |  | 2.87E-10 |  | 0.022 |  | 2.78E-14 |
|  | LTBI-HIV/LTBI |  | 0.557 |  | 4.70E-10 |  | 1.27E-09 |  | 1.84E-06 |
|  | OD/OD-HIV |  | 0.080 |  | 0.177 |  | 0.023 |  | 0.003 |
| GSE107991 | TB/Control | 1.18E-05 | 0.001 | 2.20E-06 | 1.52E-05 | 7.74E-05 | 1.18E-04 | 2.16E-06 | 3.50E-05 |
|  | TB/LTBI |  | 6.16E-06 |  | 6.16E-06 |  | 6.60E-04 |  | 6.16E-06 |
|  | LTBI/Control |  | 0.349 |  | 0.645 |  | 0.249 |  | 0.645 |
| GSE107994 | TB/Control | 6.71E-05 | 4.32E-05 | 1.60E-13 | 4.29E-08 | 1.15E-10 | 4.32E-05 | 3.24E-12 | 2.81E-07 |
|  | TB/LTBI |  | 0.003 |  | 1.91E-14 |  | 7.48E-12 |  | 4.15E-13 |
|  | LTBI/Control |  | 0.048 |  | 0.019 |  | 0.394 |  | 0.169 |
| GSE101705 | TB/LTBI | NA | 4.48E-04 | NA | 2.44E-06 | NA | 1.82E-06 | NA | 4.24E-07 |
| GSE42825, GSE42826, GSE42830 | TB/Control | 3.37E-23 | 4.74E-14 | 1.05E-33 | 2.75E-17 | 3.63E-16 | 1.12E-11 | 1.13E-34 | 2.75E-17 |
|  | TB/Active SARC |  | 0.006 |  | 1.77E-04 |  | 0.123 |  | 2.08E-06 |
|  | TB/Non-active SARC |  | 7.02E-07 |  | 9.67E-10 |  | 3.34E-05 |  | 3.92E-12 |
|  | TB/Cancer |  | 0.108 |  | 1.77E-07 |  | 1.77E-07 |  | 5.59E-04 |
|  | TB/PNA |  | 0.525 |  | 1.10E-04 |  | 6.39E-05 |  | 0.693 |
|  | Active SARC/Control |  | 7.49E-10 |  | 4.80E-17 |  | 1.23E-09 |  | 1.83E-16 |
|  | Active SARC/ Non-active SARC |  | 0.005 |  | 2.57E-04 |  | 5.59E-04 |  | 2.08E-06 |
|  | Active SARC/Cancer |  | 0.523 |  | 0.042 |  | 1.80E-05 |  | 0.775 |
|  | Active SARC/PNA |  | 0.005 |  | 0.518 |  | 0.001 |  | 5.56E-04 |
|  | Non-active SARC/Control |  | 0.033 |  | 1.19E-05 |  | 0.264 |  | 1.66E-04 |
|  | Non-active SARC/Cancer |  | 0.002 |  | 0.143 |  | 0.246 |  | 1.54E-04 |
|  | Non-active SARC/PNA |  | 9.78E-05 |  | 0.017 |  | 0.628 |  | 1.05E-08 |
|  | Cancer/Control |  | 1.69E-07 |  | 5.28E-08 |  | 0.376 |  | 1.05E-08 |
|  | Cancer/PNA |  | 0.085 |  | 0.264 |  | 0.507 |  | 0.005 |
|  | PNA/Control |  | 3.09E-07 |  | 3.40E-08 |  | 0.856 |  | 6.98E-09 |
| **FDR**: False Discovery Rate | | | | | | | | | |
| **TB:** Tuberculosis (without HIV) | | | | | | | | | |
| **HIV:** Human Immunodeficiency Virus | | | | | | | | | |
| **TB-HIV:** Tuberculosis with HIV | | | | | | | | | |
| **LTBI:** Latent tuberculosis infection (without HIV) | | | | | | | | | |
| **LTBI-HIV**: Latent tuberculosis infection with HIV | | | | | | | | | |
| **OD:** Other diseases (without HIV) | | | | | | | | | |
| **OD-HIV:** Other diseases with HIV | | | | | | | | | |
| **SARC:** Sarcoidosis (without HIV) | | | | | | | | | |
| **Cancer:** Lung cancer (without HIV) | | | | | | | | | |
| **PNA:** Pneumonia (without HIV) | | | | | | | | | |
